# Advances in Conductive Nanomaterials for Cardiac Arrhythmia and Future Directions in Bioevaluation Strategies

**DOI:** 10.1002/adhm.202503686

**Published:** 2025-11-05

**Authors:** Sumithra Y. Srinivasan, Anna Laromaine

**Affiliations:** ^1^ Institut de Ciència de Materials de Barcelona (ICMAB‐CSIC) C/ dels Til.lers s/n Campus Universitari Bellaterra Barcelona 08193 Spain

**Keywords:** biomaterials, *C. elegans*, cardiac arrhythmia, cardiac tissue engineering, conducting nanomaterials, myocardial infarction, small animals

## Abstract

Cardiac arrhythmia (CA), characterized by irregular heart rhythms, affects nearly 90% of individuals with cardiovascular diseases. Commonly triggered by myocardial infarction (MI) or ion channel dysfunctions, CA is traditionally treated using pacemakers, cardioversion, ablation, and anti‐arrhythmic drugs. Recently, emerging strategies like cardiac patches and injectable formulations with conductive nanomaterials (CNMs) have shown promise in restoring cardiac rhythm post‐MI. This review explores CNMs—such as gold nanoparticles, carbon nanotubes, and conjugated polymers—that mimic the electrical and mechanical properties of native cardiac tissue. While in vitro studies show encouraging results, translating CNMs to clinical settings faces challenges. Few studies have assessed their safety and efficacy in rodent models, and none in larger animals. This gap stems from the complexity and ethical hurdles of large animal research. To address this, the review advocates using small animal models like *zebrafish, Drosophila melanogaster, and Caenorhabditis elegans*. These models offer insights into pharmacokinetics, pharmacodynamics, genetic effects, and cardiac parameters such as ejection fraction and cardiac output—data often unattainable in vitro. Such platforms can better evaluate CNMs' safety and efficacy than 2D/3D cultures, accelerating progress toward clinical application.

## Graphical abstract

Conductive nanomaterials (CNMs) show promising potential in cardiac tissue regeneration and restoring cardiac rhythm as reported in several in vitro studies. This review article elaborates on the applications of CNMs in the treatment of myocardial infarction (MI) and MI‐induced cardiac arrhythmias, and it sheds light on the innovative approach of utilising small animal models for CNM evaluations, to accelerate their progress from bench‐to‐bedside.

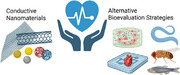

## 1 Introduction

Cardiovascular diseases (CVDs) affect the circulatory system and the heart. CVDs, with 32% global deaths and 45% in Europe, still stand as the top contributor to mortality worldwide.^[^ ^1^, ^2^ ^]^ CVDs primarily encompass conditions such as coronary artery disease, valvular disorders, aneurysms, cardiac arrhythmias, cardiomyopathy, pericarditis, and heart failure. Among these, cardiac arrhythmia (CA) is notably prevalent, with 90% of cardiac patients exhibiting arrhythmia symptoms, contributing to 18% of deaths associated with heart conditions.^[^ ^3^, ^4^ ^]^ CA is characterized by irregular heart rhythms resulting from improper electrical signal transmission across cardiac cells.^[^ ^5^ ^]^ This electrophysiological irregularity can manifest as tachycardia, characterized by a heart rate exceeding 100 beats per minute, or bradycardia, where the rate falls below 60 beats per minute. Tachycardia is further classified into two types based on its origin: supraventricular arrhythmias arise from the atria or atrioventricular node (AVN), while ventricular arrhythmias originate in the ventricles.^[^ ^6^ ^]^

**Figure 1:**
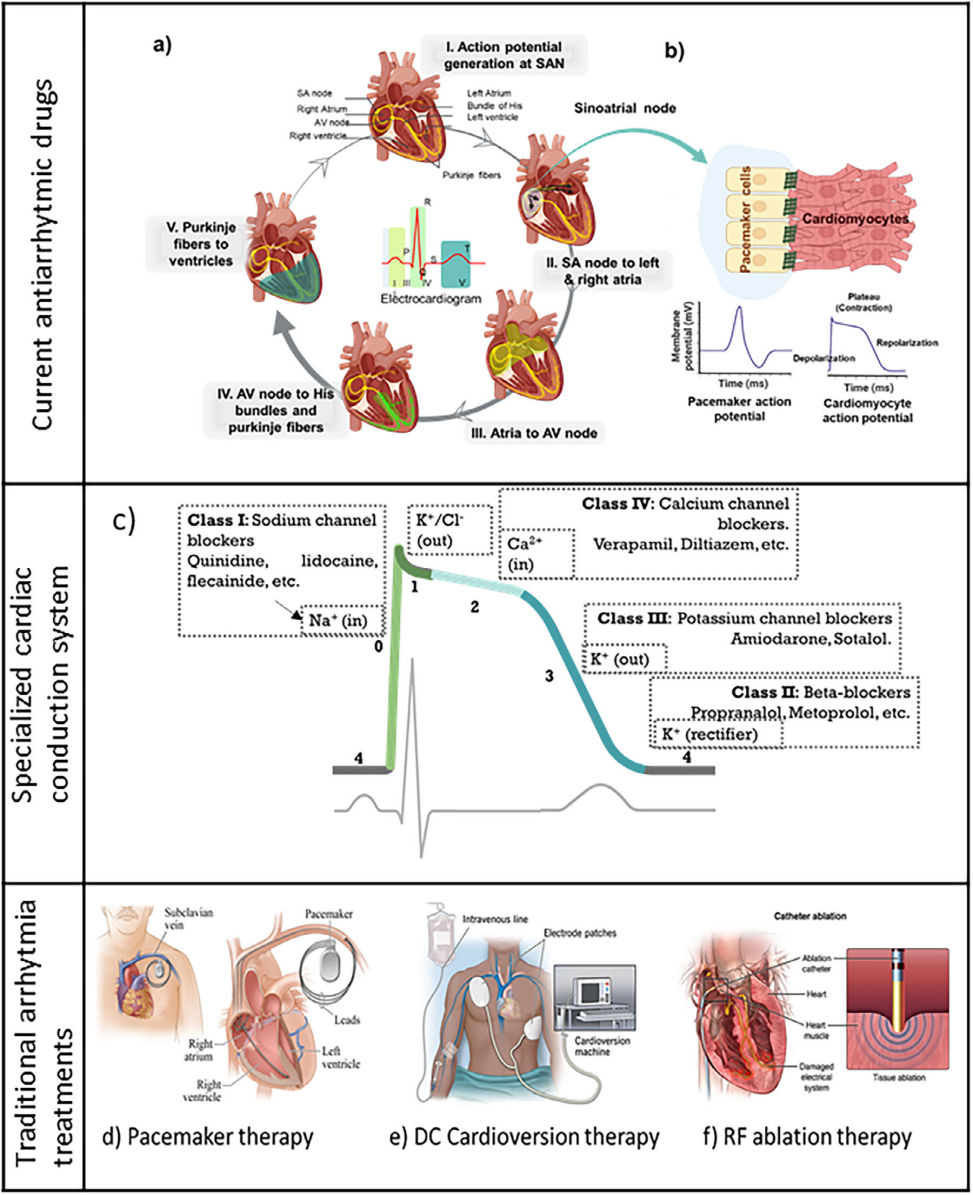
a) The cardiac action potential propagation across the heart originates from the sinoatrial node. It spreads to the ventricles via the atria, the AV node, the His bundles, and the Purkinje fibers. At the center, the electrocardiogram illustrates the temporal phases of cardiac conduction, with the P wave representing atrial depolarization, the QRS complex corresponding to ventricular depolarization, and the T wave indicating ventricular repolarization. b) Depicts the interaction between pacemaker cells and cardiomyocytes at the sinoatrial node (SAN), which is mediated by gap junctions, causing depolarization and repolarization. The respective action potentials of pacemaker cells and cardiomyocytes are shown below. c) According to the Vaughan Williams classification, this is a description of current antiarrhythmic drugs acting at different stages of the human action potential (top) and ECG (bottom). Last row, traditional arrhythmia treatments: d) pacemaker therapy – the pacemaker with the leads connected to the heart, e) DC cardioversion therapy – electrode patches deliver the electric current to stimulate the cardiac impulse, and f) RF ablation therapy ‐the catheter is inserted into the atria to deliver the RF energy. Reproduced and adaptedwith permission^[^ ^11^ ^]^ 2023 UAB.

The heart comprises a specialized cardiac conduction system (CCS), made of the sinoatrial node (SA node), atrioventricular node (AVN), His bundles, and Purkinje fibers, in addition to atrial and ventricular myocardium. The electrical signal initiates at the SA node and propagates to the left and right atria before propagating through the AVN, His bundles, and Purkinje fibers. Here, the signal is delayed for prolonged contraction of the atria to ensure complete blood transport to the ventricles, before ventricular contraction (**Figure** **1**a). This CCS is responsible for muscle contractions, ensuring consistent rhythmic beating of the atria and ventricles to facilitate blood circulation.^[^ ^7^, ^8^ ^]^ While CA can arise due to multiple causes like electrolyte imbalances, medications, etc., the two major causes are disruption in the CCS by myocardial infarction (MI) and/or dysregulation of ion channel subunits. In MI, ischemic injury leads to necrosis of myocardial tissue, disrupting the integrity of the cardiac conduction pathways, causing aberrant electrical activity_,_^[^ ^9^ ^]^ resulting in arrhythmias. Alternatively, dysfunction in cardiac ion channels, which mediate intercellular electrical coupling and action potential propagation, can disrupt electrophysiological homeostasis, predisposing the myocardium to arrhythmogenic events.^[^ ^10^ ^]^

Within the CCS, the propagation of electrical impulses is mediated by specialized interactions between pacemaker cells located in the sinoatrial node (SAN) and cardiomyocytes, as well as between different types of cardiomyocytes, which are cardiac‐specific contractile muscle cells situated in the atrial and ventricular muscles.^[^ ^8^ ^]^ These cardiomyocytes are connected to pacemaker cells and to each other via gap junctions (Figure 1a **),** which consist of ion channels that facilitate the movement of cations such as Na^+^, Ca^2+^, and K^+^. The voltage‐gated calcium channels respond to the electrical impulse generated at the SAN, prompting an influx of Ca^2+^, further resulting in a sequence of cation influx and efflux. This transmembrane movement of cations alters the membrane potential, resulting in depolarization and repolarization phases. Consequently, these ion channels are crucial for the electrical coupling of cells and the propagation of action potentials, which are essential for the synchronized contraction of the heart muscle.^[^ ^8^ ^]^ Therefore, any disruption in the CCS due to ion channel malfunctions, MI, congenital defects, or developmental issues can lead to arrhythmias.

## 2 Traditional Cardiac Arrhythmia Treatment Strategies

Current methodologies for arrhythmia intervention include pharmacological and external or surgical therapies. Pharmacological strategies involve administering antiarrhythmic medications that target ion channels affecting cardiac rhythm. External interventions include pacemaker implantation, direct current (DC) cardioversion, and radio frequency (RF) ablation.^[^ ^12^ ^]^

In pharmacological therapy, anti‐arrhythmic drugs are usually employed to address the overactivation of ion channels in the case of tachyarrhythmias.^[^ ^13^ ^]^ These therapies are categorized according to the specific ion channels and the stage of the action potential by Vaughan–Williams classification (Figure 1c),^[^ ^6^, ^12^ ^]^ namely, sodium channel blockers (class I), beta‐blockers (class II), potassium channel blockers (class III), calcium channel blockers (class IV), and class V drugs, which are drugs that do not target ion‐channels or whose mechanisms are not yet known. Despite their widespread use in arrhythmia treatment, these drugs have been reported to cause significant side effects, including heart failure, stroke, obstructive lung disease, headaches, and contraindication, causing unexpected bradycardia while attempting to treat tachycardia.^[^ ^13^, ^14^ ^]^

Pacemakers are also commonly used to manage bradycardia and certain types of tachycardia, syncope, and heart failure.^[^ ^15^ ^]^ Pacemakers consist of leads that connect the device to the heart (Figure 1d) to establish a connection and regulate cardiac rhythm. They can generate action potentials and propagate electrical signals to initiate depolarization and therefore contraction.^[^ ^16^ ^]^ Pacemakers are useful to manage conditions like sinus bradycardia, SAN dysfunction, and atrioventricular (AV) blocks, where stimulation is necessary.^[^ ^17^ ^]^ Pacemaker therapy is one of the most widely implemented interventions after anti‐arrhythmic drugs, with almost 900000 devices implanted globally each year. However, several limitations are associated with pacemakers – the procedure is inherently invasive, requiring surgery, and the device is susceptible to malfunction due to lead displacement, lead failure, etc. Additionally, the limited battery lifespan – typically 10–15 years‐ necessitates continuous monitoring, increasing the long‐term maintenance costs. Moreover, serious adverse effects may occur, such as hematoma, pneumothorax, myocardial perforation, immune rejection, and thrombosis.^[^ ^18^, ^19^ ^]^

On the other hand, DC cardioversion treatment employs an external electric shock applied via electrode patches to the heart restoring disordered electrical conduction and enable the SAN to regulate heart rate (Figure 1e). Unlike pacemakers, DC cardioversion therapy is non‐invasive; nevertheless, long‐term co‐administration of antiarrhythmic drugs is required to maintain the restored functions, posing the risk of arrhythmia recurrence. DC cardioversion therapy also poses risks of embolus and thrombus formation, thereby increasing the risk of sudden cardiac death and stroke.^[^ ^12^ ^]^

Among the external therapies, radio frequency (RF) ablation represents a significant advancement in treating and managing arrhythmias. This procedure involves the insertion of an electrode into the heart via a catheter. The electrode precisely maps the heart's electrical activity to identify the tissues where arrhythmias originate, which are then ablated by radio frequency energy (Figure 1f). RF energy is a form of electromagnetic radiation that uses high‐frequency electrical currents to heat and destroy target tissues. Destruction of the affected tissue mitigates the risk of pro‐arrhythmic effects in the future, reducing the need for continued administration of anti‐arrhythmic drugs. Owing to this, RF ablation poses fewer side effects compared to other external therapies. Nevertheless, some complications are also reported, including bruising or bleeding arising from the catheter's insertion, infection, and blood clots.^[^ ^20^ ^]^

## 3 Nanomedicine Approaches to Arrhythmia Therapy

The rise of nanotechnology has significantly transformed theragnostic research since the 2000s. Especially, the use of nanomaterials in the diagnosis, treatment, and management of CVDs has tremendously increased, with predominantly rapid growth observed over the past decade (2015–2025)^[^ ^21^ ^]^ from 200 publications in 2015 to 1800 in 2024 (Data obtained from the Web of Science database, excluding review articles and book chapters. Search (April 2025) Criteria: (TS (topic search) = (nanoparticle* or nano* or nanotechnology* or nanomedicine* or nanosheet* or nanofiber* nanorod* or nanotube* or nanowire* or nanovesicle*)) AND TS = (cardiovascular disease* or arrhythmia* or infarction* or heart failure* or heart* or valve disease* or coronary artery disease* or valve aneurysm* or atherosclerosis* or cardiomyopathy* or pericarditis*). In the context of arrhythmia treatment, nano‐biomaterials find a multitude of applications as: i) drug delivery vehicles, minimizing off‐target toxicity; ii) bioimaging agents, enabling early diagnosis and prevention of heart failure; and iii) tissue engineering scaffolds, facilitating cardiac regeneration and healing.^[^ ^22^, ^23^ ^]^ Notably, over the past five years, there has been a significant and consistent increase in research interest in these biomaterials, since around 10% of the 2024 articles also included biomaterials as a keyword. (Same search criteria as before, however, including biomaterials).

### 3.1 Nanoparticle Assisted Drug Delivery

**Figure 2:**
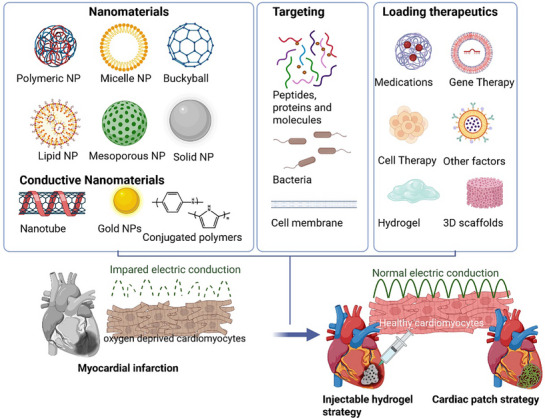
Illustration summarizing current nano‐pharmacology strategies for cardiac therapies, showing examples of nanoparticles used, surface modifications, and cargo materials usually loaded into the NP carriers for cardiac delivery. Reproduced under the terms of the CC‐BY license. 2023, Li et. al. published by MDPI.^[^ ^24^ ^]^ The bottom part depicts cardiomyocytes in the infarcted region and their impaired electric conduction, some nano‐pharmacology strategies for cardiac therapies, indicating the target of the therapy, cardiomyocytes, and improving electrical conduction. Novel strategies currently also combine the therapeutics and targeting moieties in hydrogels or scaffolds, resulting in suturable cardiac patches or injectable hydrogels for MI repair. The figure is created with BioRender.com, adapted with permission. ^[^ ^24^ ^]^ 2023, MDPI, and ^[^ ^11^ ^]^ 2023, UAB.

Nanoparticles (NPs) are popular as drug carriers owing to their nanoscale dimensions, enabling targeted drug delivery within the heart. Various nanoparticles, including polymeric, nanotubes, mesoporous, and lipid nanoparticles, have been identified as effective carriers for cardiac drugs^[^ ^22^, ^23^, ^24^ ^]^ (**Figure** **2**). The high surface area of NPs allows surface modifications to change the surface charge, conjugate ligands, etc., which can enhance cell‐nanoparticle interactions (electrostatic or biochemical) and improve cellular uptake. The surfaces of NPs are often conjugated with targeting moieties, such as cardiac‐targeting peptides and membrane‐binding proteins, which facilitate precise delivery, enhance therapeutic efficiency, and thereby mitigate off‐target side effects. The unique properties of materials at the nanoscale have been studied for the delivery of anti‐arrhythmic drugs for pharmacological therapy, cardiomyocytes and stem cells for regeneration, and miRNA for gene therapy.^[^ ^24^ ^]^

For instance, modifying the surface of cyclodextrin NPs loaded with amiodarone with L‐lysine facilitated the targeted delivery to cardiac tissue‐resident macrophages and improved the drug's efficacy in synchronizing cardiac rhythm.^[^ ^25^ ^]^ The nanocarrier encapsulation resulted in a 250% increase in the heart´s uptake compared to the lungs and unencapsulated amiodarone, which exhibited prolonged circulation times and off‐target accumulation in the thyroid, liver, and lungs. In another recent work,^[^ ^26^ ^]^ researchers developed polyethylene imine (PEI) coated gold nanoparticles embedded in collagen hydrogel. The PEI coating imparted a positive charge to the surface of the NPs, facilitating effective conjugation with the negatively charged drug, phenylephrine, through electrostatic interaction. The PEI‐coated NPs‐hydrogel‐drug complex resulted in an earlier onset of synchronous contractions, shorter contraction duration, relaxation time, and a higher contraction amplitude, compared to the drug loaded onto collagen hydrogels without the surface‐modified NPs, underscoring the role of NPs in efficient drug release and reduced side effects.

### 3.2 RF Ablation with Nanoparticles

The conventional RF ablation strategy risks can damage bystander cells. Whereas nano‐ablation therapy uses photosensitizer nanoparticles (NPs) to focus RF energy and facilitate myocyte‐specific ablation only in the affected regions. This approach has been investigated by Avula et al.^[^ ^27^ ^]^ who employed chlorin e6 (Ce6) photosensitizer modified with polyethylene glycol (PEG), and surface modified with cardiac targeting peptide (CTP) molecules. The ablation through photosensitizer‐NP‐CTP complex resulted in complete electrical blockage at the ablated region, and restored the cardiac rhythm in rat and sheep hearts. Thus, in this work, the authors achieved cell‐specific ablation of cardiomyocytes with the help of CTP‐modified PEG Nanoparticles while surrounding cells were completely unaffected. Metallic nanoparticles such as copper (Cu), iron oxide (FeO), and titanium (Ti) are also promising candidates employed in the catheter tips in nano‐ablation therapies.^[^ ^28^ ^]^ Metallic NPs possess electrical and thermal conductivity, which enables them to receive and propagate the incoming RF energy. This results in deeper penetration and more intense thermal injury at the affected regions than ablation treatment without NPs.

## 4 Conducting Nanomaterials in Cardiac Arrhythmia Treatment

Cardiac muscles are inherently electrically conducting. Whereas after MI, ischemia and necrosis result in dead cardiomyocytes, which are replaced by non‐conductive fibrotic scar tissue. Furthermore, several reports suggest that the surviving cardiomyocytes in and around an infarct exhibit impaired conduction even if they are viable^[^ ^29^, ^30^ ^]^(Figure 2). Electrically conducting nanomaterial around the infarcted tissue facilitates the conduction flow of cardiac impulses, restoring cardiac rhythm. In the past decade, using conductive nanomaterials to address MI and MI‐induced arrhythmias has been gaining attention.^[^ ^31^ ^]^ Contemporary researches are increasingly directed toward the use of nanocomposites composed of polymeric matrices or injectable hydrogels with conducting nanomaterials, often incorporating cardiomyocytes or stem cells, to support cardiac regeneration and healing of the infarcted area (Figure 2). Nanomaterials with electrical conductivity, such as conjugated polymers, gold nanoparticles, and carbon nanotubes, are frequently employed in these applications (Figure 2).^[^ ^32^ ^]^ This area of research has recently gained attention, with surging research studies worldwide over the past few years.^[^ ^31^ ^]^

### 4.1 Carbon Nanotubes

Carbon nanotubes (CNTs) are 1D graphene sheets that are rolled to form hollow nanotubes with diameters in the nanoscale range and lengths in microns, exhibiting high electrical conductivity in the range of 10^3^–10^5^ Scm^−1^. The dimensions of CNTs resemble the fibrous components of the extracellular matrix (ECM) of the cardiovascular system.^[^ ^33^ ^]^ CNTs are recognized as one of the strongest materials available, with tensile strengths reaching up to 1 TPa and stiffnesses up to 100 GPa, which is particularly advantageous in cardiac tissue engineering due to the repetitive contractile forces involved. Furthermore, the one‐dimensional rod‐like shape of CNTs aids in promoting the orientation of cardiomyocytes and imparts directionality, making them well‐suited for CTE applications.^[^ ^33^, ^34^, ^35^ ^]^ It is noteworthy that the biocompatibility and hydrophilicity of CNTs can be finely adjusted by altering the solvents and surfactants used during synthesis, as well as by modifying physical properties such as the number of walls (single or multi‐walled), length, aspect ratio, and by conjugating different functional groups to change the chemical composition.^[^ ^36^, ^37^ ^]^ For example, CNTs are often covalently functionalized with carboxyl groups to enhance their hydrophilicity and dispersion in aqueous solvents for biomedical applications.^[^ ^36^ ^]^ Additionally, CNTs are frequently combined with other biomaterials, particularly biopolymers such as gelatin, to improve biocompatibility.

Gelatin methacryloyl (GelMA) is a methacrylate‐modified denatured collagen derivative recognized as a significant scaffold material for tissue engineering applications. In this regard, Shin et al.^[^ ^38^, ^39^ ^]^ reported that incorporating CNTs into the GelMA matrix enhanced its mechanical and electrical properties and improved cell adhesion, maturation, and cell‐cell electrical communication among neonatal rat cardiomyocytes. The authors have thoughtfully studied different concentrations of CNTs (1, 3, and 5 mg/mL) in 5% GelMA, and it appears that the mechanical, structural, and electrical properties, along with cellular behaviors such as biocompatibility, adhesion, and maturation, are influenced by the CNT concentration. Interestingly, CNT‐GelMA also offers protection against tissue damage caused by cardiotoxic (doxorubicin) drugs and cardiac inhibitors (heptanol) by acting as free‐radical scavengers. A subsequent study by Sun et al.^[^ ^40^ ^]^ attempted to gain insights into the underlying mechanism of cardiac cell behavior in CNT‐GelMA materials using immunohistochemistry and calcium measurements. The authors postulated that the increased intracellular Ca^2+^ transient levels in the presence of CNT‐GelMA may be attributed to enhanced cell–cell coupling due to the material's conductivity. They also observed that CNTs play a significant role in the assembly and formation of intercalated discs (IDs) between cardiac cells, thereby enhancing gap junction and mechanical junction formation. Similarly, Lee et al.^[^ ^41^ ^]^ reported an enhancement in integrin‐mediated mechanotransduction among cardiomyocytes seeded on CNT‐GelMA scaffolds. They also compared cell behavior with other carbon‐based nanoparticle‐GelMA scaffolds, such as graphene oxide (GO) (non‐conductive) and rGO (conductive)‐GelMA materials. This study revealed that CNT‐GelMA provides the most native ECM‐like microenvironment and nanofibrous architecture, enabling better cell maturation and cardiac marker expression, in addition to more ventricular‐like phenotypes observed in cells grown on CNT‐GelMA substrates, unlike the GO and rGO counterparts. This study establishes that not only the chemical composition but also every property of the material, such as shape, morphology, surface roughness, and conductivity, significantly influences the mechanical and electrical cues to the cardiac cells, thereby affecting the interaction mechanism between the cells and the surfaces.

In the realm of studying various scaffold morphologies, Bagheri et al.^[^ ^42^ ^]^ recently designed a 3D nano‐biohybrid system featuring CNT forests coated with gelatin for cardiac tissue culturing. The scaffolds exhibited bamboo‐like nanostructures with vertical, wavy, and entangled CNTs, complemented by a thin gelatin coating on the surface of the CNT forest. The application of the gelatin coating, followed by incubation in a cell culture medium, resulted in an almost twofold increase in conductivity (from 0.3 to 0.6 Scm^−1^). Both fibroblasts (NIH 3T3 mouse cell line) and primary rat cardiomyocytes have been shown to adhere to the top and side walls of CNT forest islands, forming a 3D network of living cells. The fibroblasts demonstrated a viability of 89% on day 3, while cardiomyocytes exhibited an 89% viability on day 1, with no significant difference in viability compared to the control up to day 11, indicating that the scaffolds did not exhibit toxicity to either cell type. Furthermore, the authors observed that the scaffolds did not influence the cytoskeleton, adhesion, migration, or spreading of fibroblasts, as evaluated by RT‐PCR. Similarly, in cardiomyocytes, the expression levels of cardiac markers α‐actinin and troponin‐T were examined using confocal microscopy, and the genes involved in cardiomyocyte contraction and cell–cell communication showed no significant difference compared to controls. Thus, CNT‐gelatin scaffolds, with their biocompatibility, controllability through electrical signaling, and unique 3D morphology, present a promising opportunity for future bioelectronic systems for cardiac tissues.

**Figure 3:**
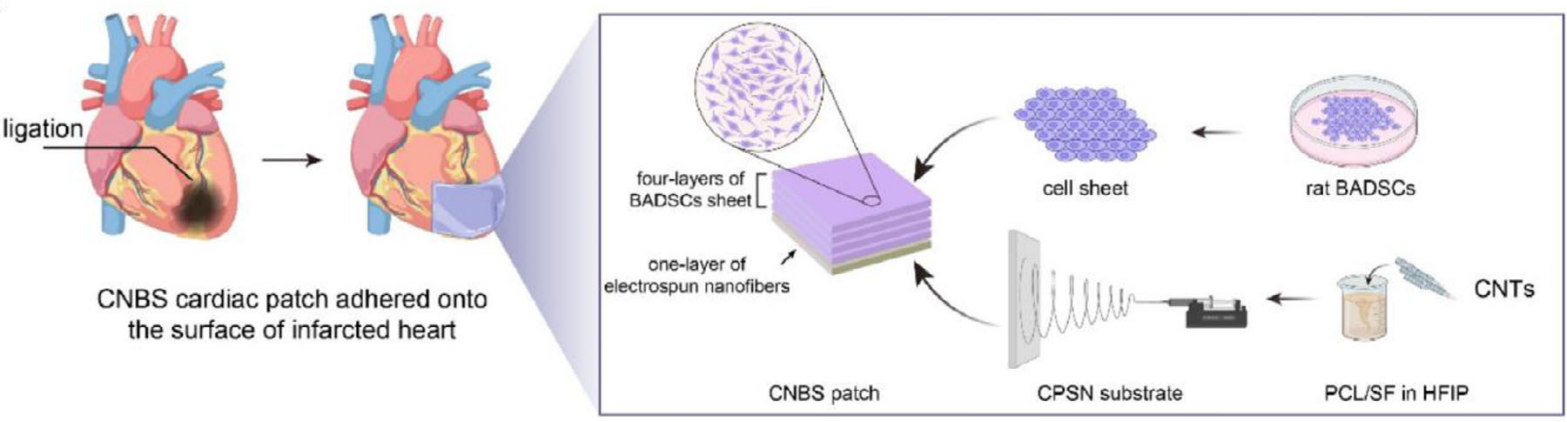
Schematic illustration of the multi‐layered cardiac patch consisting of a stem cell sheet system (BADSCs) and a biomaterial made of CNT‐PCL‐SF nanofibers, and its application for the treatment of MI. Reproduced with permission. ^[^ ^43^ ^]^ 2023, KeAi Publishing.^[^ ^43^ ^]^

In a recent study,^[^ ^43^ ^]^ researchers designed a novel multi‐layered cardiac patch (**Figure** **3**) using a combination of a biomaterial substrate and a stem cell sheet system for the treatment of MI in rats. The patches are composed of brown adipose‐derived stem cell sheets (BADSCs), which contribute to cell regeneration and repair of the infarct, and are skillfully combined with a biomaterial made of multi‐walled CNTs‐containing electrospun polycaprolactone (PCL)/silk fibroin (SF) nanofibers (CPSN), providing mechanical strength. Immunofluorescence staining confirmed the differentiation of BADSCs into cardiomyocytes through the elevated expression of CX43, α‐actinin, and CTnT. Furthermore, RT‐PCR assays revealed a two‐fold increase in the expression of VE‐cadherin and N‐cadherin compared with cells cultured in suspension, suggesting enhanced cell motility and vascularization, respectively. The authors also reported an increased intracellular calcium influx, signifying cardiomyogenesis, and upregulated M2 macrophages, which indicate augmented myocardial regeneration. In summary, cardiac patches have demonstrated elevated levels of pro‐angiogenic, pro‐migratory, and anti‐inflammatory factors compared to cells in suspension, resulting in improved cardiac remodeling and, consequently, repair of the infarcted heart.

### 4.2 Gold Nanoparticles

Gold nanoparticles (AuNPs) are often utilized in cardiac therapy applications because of their highly biocompatible nature as metallic conducting nanoparticles. Their highly reactive surface chemical properties make them excellent candidates for combination with other nanomaterials to create multifunctional nanocomposites with tailored properties.^[^ ^44^ ^]^ AuNPs possess unique shape‐ and size‐dependent optical absorbance and cytotoxicity,^[^ ^45^ ^]^ and they can be fashioned into various shapes, allowing for diverse applications such as nanocarriers for drug delivery and imaging for diagnostics and prognostics. Notably, although CNTs and conjugated polymers are more frequently used as cardiac scaffolds because of their high mechanical strength, ability to mimic the extracellular matrix (ECM), and provision of structural support and porosity, AuNPs are often favored for injectable hydrogels or sprayable formulations. This preference is attributed to their small size, aqueous dispersibility, and stability, which facilitate uniform particle distribution, thereby enabling consistent application on surfaces.

In a corresponding study,^[^ ^46^ ^]^ the researchers developed a surface of AuNPs engineered with custom‐designed peptides, formulated as a sprayable solution for MI healing. The study evaluated the in vivo efficacy of these nanoparticles in mice induced with MI. The cardiac strain force in mice treated with peptide‐grafted AuNPs was comparable to that of SHAM mice, demonstrating greater contractility than that of the other treatment groups. Additionally, the ECG signal profile of mice treated with peptide‐AuNPs closely resembled that of SHAM mice, whereas the other treatment groups exhibited significant differences. The infarct size was notably reduced (≈10%) in the treated mice 28 days post‐treatment compared to that in the untreated mice (approximately 60%). The researchers hypothesized that the reduction in infarct size may be attributed to increased angiogenesis, wound‐healing macrophages, gap junction formation, and intercellular communication. The on‐site spray application of the nanoparticles allowed for significantly smaller dosages (2.4 µg k^−1^g body mass) compared to other AuNP studies (several mg k^−1^g body mass). Furthermore, peptide grafting ensured reduced off‐target toxicity, as confirmed by the researchers through comparisons with nonprotected AuNPs, which showed infiltration into the lymph nodes and liver, thereby offering superior therapeutic quality compared to traditional scaffold designs and bare AuNPs. However, this study did not elucidate the exact mechanism of action by which the material facilitates infarct healing or the role of the conductivity of AuNPs in electrical signal propagation. Further exploration of these aspects could advance this promising material toward larger animal models and clinical studies for MI healing in the future.

In contrast to the aforementioned study, where cell sheets were employed, another previously discussed study^[^ ^26^ ^]^ explored a cell‐free approach for cardiac healing using a collagen hydrogel containing PEI‐coated AuNPs loaded with phenylephrine (PL). The authors reported an improvement in cell‐cell electrical coupling and enhanced intracellular Ca^2+^ flow imparted by the conducting material (AuNPs). The PL‐loaded AuNP‐hydrogels demonstrated the capability to promote the early onset of synchronous contractions on day 2, compared to day 7 for unloaded hydrogels. Notably, the drug‐loaded hydrogels, in the absence of AuNPs, also induced arrhythmic beating. Therefore, the combinatorial effect of a drug, conducting AuNPs, and a biocompatible collagen hydrogel substrate was essential for enhancing contractility and promoting cardiac healing. While cell sheet engineering offers its own benefits, such as enhanced regeneration, a cell‐free formulation appears highly promising because of its reduced immunogenicity and tumorigenicity, as well as its flexibility, scalability, and reduced cost in terms of production, transport, and storage.

Recent research has investigated the role of gold nanorods (GNR) in the development of human‐engineered cardiac tissue (hECT).^[^ ^47^ ^]^ GNR‐fibrin hydrogel‐Matrigel composites were created to serve as a conductive extracellular matrix (ECM) for developing 3D hiPSC‐CM hECT constructs. Although the presence of GNR delayed spontaneous contractions (16 days compared to 6 days without GNR), it significantly enhanced survival, extending up to 9 months when the experiment concluded, whereas without GNR, survival was limited to 1 month post culture. The contraction force and beating frequency also increased in GNR‐hECT, peaking on day 12. While the contraction force began to decrease at day 16 for hECT without GNR, it consistently increased for GNR‐hECT, demonstrating robust beating even at day 31, day 45, and up to 9 months after treatment. Calcium imaging further revealed an enhanced mean width and more uniform Ca^2+^ transients than in hECTs without GNR. Additionally, the increased intracellular Ca^2+^ and stronger contractions indicated improved Ca^2+^ handling in the GNR‐hECTs. Finally, the GNR‐hECTs exhibited more elongated and aligned sarcomeres after nine months, closely resembling mature myocardial tissues. Consequently, the incorporation of conducting GNRs into hECTs improved the overall cardiomyocyte properties, suggesting potential future applications of conducting materials in ECM fabrication.

To thoroughly elucidate the role of conductivity in cardiac tissue engineering, a recent study^[^ ^48^ ^]^ developed hECTs using GelMA hydrogels with either conductive GNRs or non‐conductive silica NP (SNP) and compared the behavior of hiPSC‐CMs in both materials. The cells demonstrated excellent biocompatibility and similar attachment to both surfaces. The expression of α‐actinin in sarcomeres was comparable in both hECTs; however, the sarcomeres appeared more organized and striated in cells grown on GNR‐GelMA. As anticipated, more synchronized calcium spikes and increased calcium transient intensity were observed in the cells on GNR‐GelMA, suggesting enhanced electrical coupling between the cardiomyocytes. This observation was further supported by the higher levels of gap junction protein Cx43 expression in cells on conductive scaffolds compared to those on nonconductive scaffolds. This finding indicates that conductivity significantly influences tissue signal propagation, which is mediated by the electrical coupling of cells. Interestingly, the authors conducted a comprehensive gene expression profiling of hECTs and found no significant difference in overall gene expression between the two materials. Consequently, the authors concluded that conductivity plays a role in tissue connectivity and cell–cell signaling, but it may not be the sole factor in determining the function, fate, and phenotype of hiPSC‐CMs in hECTs.

### 4.3 Conjugated Polymers

**Figure 4:**
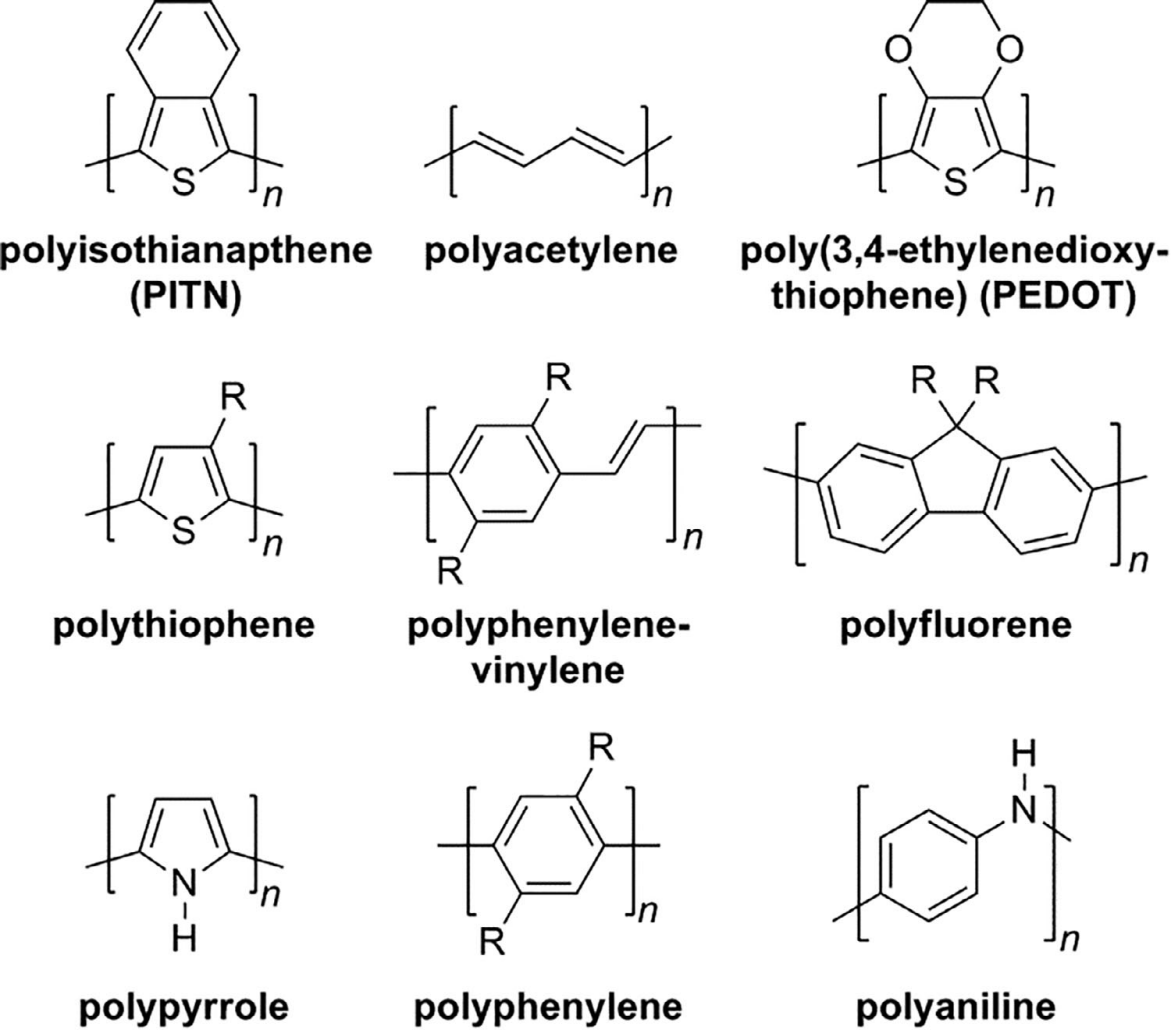
Chemical structures of some commonly used conjugated polymers. Among these, PEDOT, polypyrrole, and polyaniline are the most commonly investigated CPs for cardiac therapies. Reproduced with permission. ^[^ ^51^ ^]^ 2016, American Chemical Society.

Conjugated polymers (CPs) have alternating single and double bonds resulting in delocalized π‐electrons, which imparts its unique electrical conductivity. The nanoparticles of CPs are easy to synthesize, exhibit high aqueous and thermal stability, and can be readily functionalized at the surface with functional groups such as ─COOH, ─NH_2_, ─OH, etc., making them attractive for therapy and diagnostic applications.^[^ ^49^ ^]^ Furthermore, the conjugated structure also confers near‐infrared (NIR) absorption capacity, suitable for photodynamic, photothermal therapies, NIR imaging etc.^[^ ^50^ ^]^ This blend of features has been utilized by scientists to develop multifunctional nanomaterials for numerous applications such as cardiac therapy, smart stimuli‐responsive drug delivery, photothermal therapy, photodynamic therapy, biosensing, tissue engineering, and bioimaging. Some of the most common CPs include polypyrrole, polyaniline, polythiophene, and poly (3, 4‐ethylene dioxythiophene) (PEDOT) (**Figure** **4**).^[^ ^49^, ^51^ ^]^ In this discussion, we focus on the state‐of‐the‐art applications of the three most commonly employed CPs for treating arrhythmia: polyaniline, PEDOT, and polypyrrole nanoparticles.

#### 4.3.1 Polyaniline

Polyaniline (PANI), a polymer derived from aniline, has been extensively studied for its potential in various biomedical applications, such as tissue engineering, drug delivery, and diagnostics. PANI is noteworthy in cardiac tissue engineering because of its electrical conductivity, which can be precisely modulated through doping.^[^ ^52^ ^]^ This tunable conductivity is primarily attributed to the ability of the ‐NH group in PANI to undergo oxidation state changes via protonation and deprotonation processes. Wu et al.^[^ ^53^ ^]^ exploited the adjustable electrical properties of PANI to develop a cardiac scaffold composed of an electrospun composite of polyglycerol sebacate (PGS), PANI, and polyvinyl alcohol (PVA). In this composite, PVA was used to enhance biocompatibility, whereas PGS contributed to the elasticity of the scaffolds. Notably, increasing the PANI content not only augmented the conductivity of the patches but also enhanced the elastic modulus by nearly fivefold compared to those without PANI, although it reduced the tensile strength by approximately half, which remained adequate for cardiac tissue engineering (CTE). The scaffolds exhibited an elastic modulus ranging from 0.65 to 2.18 MPa under wet conditions and demonstrated anisotropic mechanical behavior, closely resembling that of the native myocardium. In vitro evaluation using H9c2 rat cardiac myoblast cells indicated improved cell attachment and proliferation on scaffolds containing 1.6% (w/v) PGS/PANI composite. The aligned fibrous structure of the scaffolds facilitated the elongation and alignment of H9c2 cells along the fiber direction, whereas on scaffolds with random fibers, the cells appeared rather flat. Furthermore, in vivo biocompatibility was confirmed in Wistar rats, where cells exhibited substantial infiltration and uniform migration into the pores, which was attributed to the scaffolds' elasticity, porosity, and hydrophilicity. Thus, by adjusting the PANI content and fiber alignment, scaffolds with desired electrical and mechanical properties can be developed for future applications, such as cardiac patches.

In a pioneering study,^[^ ^54^ ^]^ PANI nanoparticles (PANI NPs) were in situ polymerized on nanofibrous polycaprolactone (PCL) mats using ascorbic acid (AA) as a dopant. Traditionally, dopants such as ammonium persulfate, which are cytotoxic and require thorough removal from nanoparticles, have been utilized. In contrast, AA is biocompatible and possesses antioxidant property. In this study, antioxidant activity was conferred to the scaffolds through two mechanisms: the intrinsic antioxidant capability of PANI NPs and the additional antioxidant effect of AA. The incorporation of PANI NPs significantly enhanced the Young's modulus of the PCL scaffolds from 9 MPa to 57 MPa and increased the ultimate strength by nearly threefold. H9c2 cells cultured on these scaffolds exhibited no significant cytotoxicity until day 5. The heart inherently possesses low antioxidant properties, and excessive reactive oxygen species (ROS) production and accumulation have been reported in cardiac diseases, particularly following ischemia‐reperfusion (I/R) injury. Owing to the antioxidant properties of the scaffolds, the authors assessed cell behavior under oxidative stress induced by H_2_O_2_ and hypoxic conditions stimulated by CoCl_2_. Under H_2_O_2_ exposure, cells cultured on pure PCL demonstrated only 50% viability, whereas PANI‐AA NPs improved the viability to up to 90%. Intracellular ROS levels were also significantly reduced to nearly half in the case of PANI‐AA NP‐loaded PCL. H_2_O_2_ induces oxidative stress and aging, leading to cardiac hypertrophy, which can result in heart failure if chronic. Nuclear enlargement and plasma membrane blebbing are common features of cardiac hypertrophy. Conversely, cells on the PANI‐AA‐PCL scaffolds under oxidative stress exhibited morphology and surface areas similar to those of H_2_O_2_‐untreated cells. Furthermore, PANI‐AA NPs maintained cell survival at 80% for cells subjected to hypoxic damage induced by CoCl2. The role of PANI‐AA NPs in mitochondrial cell apoptosis was also examined by evaluating the expression of caspase‐3, a pro‐apoptotic enzyme, and Bcl‐2, an anti‐apoptotic protein crucial for membrane permeability and mitochondrial integrity. Under hypoxic conditions, cells on pure PCL showed a threefold increase in caspase‐3, whereas cells on PANI‐AA scaffolds exhibited only a 1.5‐fold increase. Similarly, Bcl‐2 levels were twice as high in cells on PANI‐AA‐PCL scaffolds under CoCl_2_ compared to those on PCL alone. The authors also tested an in vivo skin flap injury model in NMRI male mice and found that untreated injured mice exhibited 50% necrosis, whereas the patch‐treated skin flap implant showed only 10% necrosis, akin to the no implantation group. The patch also facilitated re‐epithelialization, neovascularization, and collagen synthesis, as confirmed by the increased levels of VEGF and TGF‐β. In summary, the scaffolds, with their multifunctional antioxidant nature, promoted cardiac cell adhesion and proliferation and provided essential antioxidant properties for ROS scavenging and wound healing, thereby supporting the attenuation of harmful effects after I/R injury.

Researchers have also studied conducting scaffolds as cardiac patches using true cardiomyocyte cell lines. In one such study,^[^ ^55^ ^]^ researchers prepared electrospun nanofibers of polycaprolactone (PCL) and gelatin (Ge) with PANI nanoparticles in varying ratios. Consistent with previous studies, this study also demonstrated that altering the concentrations of individual components significantly influences the resulting material properties. A higher Ge content (PCL:Ge = 40:60) enhances hydrophilicity and solubility, thereby improving solution conductivity. Various concentrations of PANI NPs were tested (0.25, 0.50, and 1.00%), and the 1% PANI NPs on PCL:Ge (40:60) were identified as the optimal candidate for maximum biocompatibility, as well as high cell adhesion, spreading, and proliferation of murine atrial cardiomyocytes. The presence of PANI NPs facilitated the formation of grouped cell cores with centers near the PANI NPs, whereas the electrical conductivity promoted stimulation and propagation of beating across cells. Interestingly, qPCR revealed that the scaffolds significantly downregulated the expression of the pro‐inflammatory cytokine TNFα, indicating potential anti‐inflammatory activity. In an interesting work by Roshanbinfar et. al.,^[^ ^56^ ^]^ the group developed fibrous mesh mats made of collagen, hyaluronic acid, and PANI (collagen‐HA‐PANi) and tested their efficacy on neonatal rat ventricular cardiomyocytes (NRVMs) and the clinically relevant hiPSC‐derived cardiomyocytes (hiPSC‐CMs) for CTE. The NRVMs showed good survival, attachment, as well as contractile ability on the mats. The presence of PANi in the mats improved the contraction duration and amplitude. The hiPSC‐CMs on these mats also attached well and spread, as confirmed by the elevated expressions of cardiac troponin I and sarcomeric α‐actinin, and exhibited improved electrical coupling as evidenced by an increase in CX43 expression. Interestingly, hiPSC‐CMs on PANi containing mats displayed faster beating frequencies, with lower amplitude, and a more regular cardiac rhythm. Similar to previous studies, modifying the levels of individual components – collagen, and PANi resulted in varying material properties. Thus, it is evident that composite nanobiomaterials, instead of single‐component materials proved to be more efficient in mimicking cardiac ECM and providing ideal properties for CTE.

#### 4.3.2 Poly(ethylenedioxythiophene) (PEDOT)

PEDOT from the polythiophene family with its π‐conjugated structure is distinguished for its superior electrical conductivity and is widely utilized in biosensors and bioelectronics applications. In contrast to other conducting polymers, PEDOT is recognized for its exceptional environmental stability, which is attributed to the ether linkages within its polymeric backbone.^[^ ^57^ ^]^ Polystyrene sulfonate (PSS) is frequently utilized as a dopant for PEDOT, particularly in biomedical applications, as PSS improves solubility and processability, enhances chemical and electrochemical stability in aqueous environments, and mitigates excessive inflammatory responses.^[^ ^58^ ^]^ Interestingly, the mechanical properties of PEDOT can also be fine‐tuned by adjusting the PSS ratio. The microstructure of PEDOT: PSS is defined by PEDOT grains encapsulated in a PSS‐rich shell owing to its hydrophilic nature. In addition to its high electrical conductivity, ethylene deoxy functional groups contribute to improved solubility, processability, and the ability to generate various nanomaterials, including composites, thin films, and coatings.^[^ ^57^, ^58^ ^]^ Particularly in cardiovascular tissue engineering (CTE), PEDOT‐based nanocoatings garner increasing research interest, as PEDOTs are well‐suited for application as surface coatings on cardiovascular stents.

Yang et al.^[^ ^59^ ^]^ conducted a study on the anti‐fouling and anti‐clogging properties of PEDOT coatings on stainless steel (SS) surfaces, focusing on their potential applications as stent coatings. This study explored the incorporation of PSS, heparin (HEP), and graphene oxide (GO) as additives with PEDOT, which was electrochemically polymerized on SS. Owing to the highly anionic nature of these materials, they exhibit strong repulsive interactions with the surrounding anionic human serum albumin in the blood, thereby preventing protein deposition and rendering the surfaces antifouling (PEDOT/GO/HEP > PEDOT/GO > PEDOT/PSS ≈ PEDOT/HEP). Hemocompatibility was evaluated based on three criteria: i) HSA adsorption, which was highest for stainless steel, reduced by 20% for PEDOT/PSS and PEDOT/HEP, by 40% for PEDOT/GO, and 42% for PEDOT/GO/HEP; ii) platelet adhesion on PEDOT‐coated surfaces was only 7% compared to adhesion on plain SS surfaces; and iii) coagulation time was significantly extended, up to five times longer, with coagulation occurring at 45 s for PEDOT/PSS, /GO, /HEP, and approximately 225 s for PEDOT/GO/HEP. The authors proposed that this may be attributed to the multilayer structure of GO, which facilitates the entrapment of heparin between the layers, further delaying blood coagulation. Furthermore, the materials demonstrated biocompatibility, with a relative growth ratio of NIH3T3 mouse embryonic fibroblasts exceeding 100.

Interestingly, while the production of scaffolds containing conjugated polymers such as PANI and Ppy is not cell‐compatible and must be seeded with cells only after post‐fabrication, PEDOT: PSS scaffolds have the unique ability that they can be produced in the presence of cells. Bearing this in mind, a fascinating study involving PEDOT: PSS containing collagen‐alginate cell‐laden hydrogels (eCA‐gels) was fabricated and tested in the presence of cardiomyocytes.^[^ ^60^ ^]^ In this work, the authors prepared a mixture of collagen I, sodium alginate, and PEDOT: PSS pre‐gelled solution, to which cardiomyocytes were resuspended before the final, second gelation of collagen I, resulting in functional 3D cell‐laden electroconductive hydrogels. The authors showed that neonatal rat cardiomyocytes survived, attached, and matured in PEDOT: PSS hydrogels, with enhanced beating frequencies of more than 200 beats/min, improved alignment as well as density, and an elevated connexin‐43 expression compared to the non‐conducting hydrogels. Additionally, hiPSC‐CMs were also successfully encapsulated and showed improved maturation and beating properties inside the PEDOT: PSS‐containing hydrogels, with a near adult sarcomeric length (2 µm), and faster contractions with higher amplitudes. This work demonstrates that PEDOT:PSS can be incorporated into hydrogels in the presence of living cardiomyocytes, supporting their survival, maturation, and functional beating, paving the way for electrically conducting 3D hydrogel‐tissue constructs.

A recent study by the same group^[^ ^61^ ^]^ utilized PEDOT: PSS within an injectable collagen hydrogel formulation to mitigate post‐infarct CA, particularly ventricular tachycardia (VT). Notably, the incorporation of PEDOT: PSS facilitated collagen gel formation and enhanced both micromorphology and conductivity. While PEDOT: PSS is anticipated to facilitate electrical signaling between cells, collagen provides domains for cell attachment. In conjunction with hiPSC‐CMs, the hydrogels promoted cardiac remuscularization, with sarcomeres achieving a near‐adult length (2 µm). The electrical coupling between two engineered cardiac tissues was examined using overnight proximity culture. Calcium flow imaging demonstrated sequential calcium flow in collagen hydrogel‐based tissue, whereas collagen‐PEDOT: PSS hydrogel‐based tissues exhibited synchronous flow, with an improved conduction velocity from 0.4 to 2.8 cms^−1^ in the presence of PEDOT: PSS. Additionally, RNA sequencing results indicated enhanced maturation through the upregulation of genes associated with electric conduction (SCN5a, HCN4, etc.), structure (MYH7, etc.), Ca^2+^ handling (CACNA1c, RYR2, CASQ2, etc.), cell‐matrix interaction (integrin subunits), and N‐cadherin, which are responsible for structural integrity, electrical coupling, and synchronous contractions. In vivo evaluation in an acute cryo‐infarcted CD1 wild‐type mouse model further confirmed the VT‐preventing capability of the hydrogels, with VT incidence post‐MI reduced to 25% compared to 90% in collagen‐only hydrogels, and improved cardiac function in the injured hearts with hiPSC‐CMs. This study underscores the promising potential of these hydrogels in post‐infarct CA prevention and warrants further investigation in large animal and patient‐relevant models in the future.

#### 4.3.3 Polypyrrole (Ppy)

Polypyrrole (Ppy) is a polymer derived from pyrrole, which is made of a heteroaromatic ring with a secondary amine group. Ppy acquires electrical conductivity upon oxidation and doping with an anion. In addition to conductivity, the aromatic structure imparts a strong absorption in the near‐infrared (NIR) region.^[^ ^62^ ^]^ Since Ppy is made conductive by doping, which leads to oxidation, the range of conductivity can be adjusted by optimizing the doping and therefore, the resulting oxidation levels.^[^ ^63^ ^]^ Ppy NPs exhibit excellent aqueous stability and are highly thermally stable up to 350 °C,^[^ ^64^ ^]^ enabling facile storage and transport, a significant factor for commercial nanomedicine applications. Despite such promising material properties, Ppy NPs are brittle, rigid materials with poor flexibility and ductility; however, they can be improved in composite formulations by combining with flexible materials. Consequently, it is often combined with hydrogel polymers for injectable formulations^[^ ^65^, ^66^ ^]^ or with polymeric membranes to create tissue engineering scaffolds.^[^ ^67^, ^68^, ^69^, ^70^ ^]^ The polymeric reinforcement could be synthetic polymers such as polylactic‐co‐glycolic acid, polycaprolactone, polyurethane, and polycarbonate or natural polymers like cellulose, alginate, and gelatin. Recently, Aziz et al.^[^ ^71^ ^]^ developed polycarbonate‐polyurethane (PCNU) fibrous scaffolds containing Ppy NPs by electrospinning. The scaffolds promoted cell alignment in isolated neonatal rat cardiomyocytes, the electrical conductivity aided in synchronizing the contractions across physically separated cardiomyocyte clusters, and restored atrial fibrillation in vivo in rat hearts. However, in scaffolds made of synthetic polymers, long‐term toxicity can be an issue, as in the case of PCNU, degradation can lead to release of CO_2_ and other toxins, such as glycols and diamines. Therefore, use of natural biopolymers like alginate,^[^ ^72^ ^]^ decellularized extracellular matrix (dECM),^[^ ^67^, ^73^ ^]^ cellulose,^[^ ^74^, ^75^ ^]^ and collagen^[^ ^76^, ^77^ ^]^ is gaining popularity because of their high biocompatibility, excellent electrical, mechanical, and structural properties.

**Figure 5:**
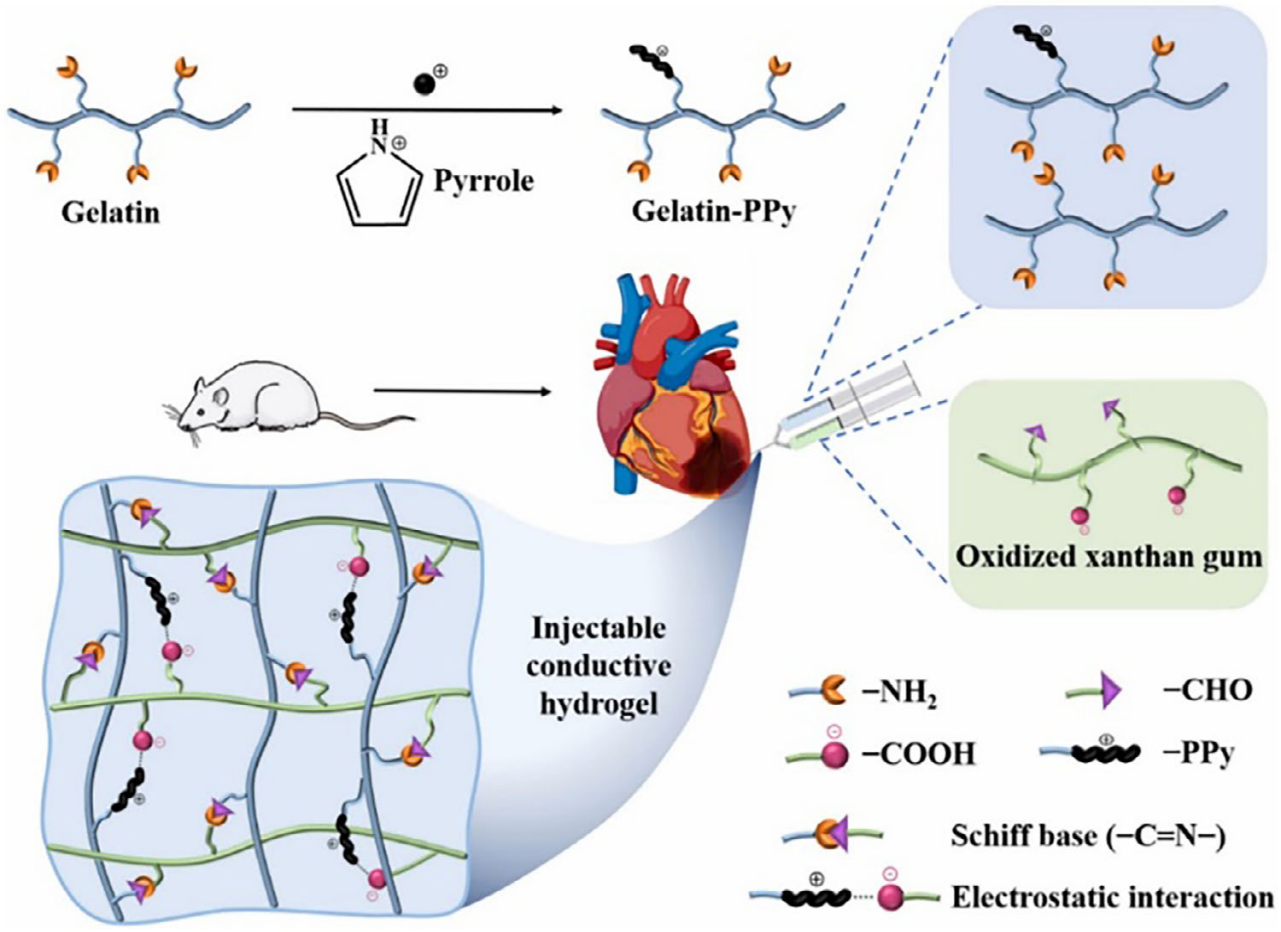
Injectable hydrogel made of gelatin‐xanthan gum containing Ppy NPs. The hydrogel restored cardiac function of infarcted rat hearts upon intracardial injection. Reproduced with permission. ^[^ ^78^ ^]^ 2023, KeAi Publishing.

Bearing this in mind, an injectable formulation using the natural biopolymer gelatin‐xanthan gum hydrogels was investigated by Zhang et al. The hydrogels were made conductive by adding Ppy NPs (**Figure** **5**). These hydrogels prevented arrhythmias in rats, enhanced the conduction velocity of impulses across the heart, reduced scar size, increased vessel density, and decreased the inflammatory response surrounding the infarcted region. Despite their porous morphologies, the microstructures of these hydrogels do not resemble the native extracellular matrix (ECM). Therefore, biopolymers that naturally exhibit a random fibrous matrix with interconnected mesh architecture, such as cellulose, may be advantageous in promoting improved cell behavior, including adhesion, cell alignment, migration, and maturation. He et al.^[^ ^75^ ^]^ fabricated Ppy NPs functionalized cellulose hydrogels, obtained from tunicate sea squirts. Evaluations on cultured primary cardiomyocytes indicated that the composite tunicate cellulose‐Ppy scaffolds resulted in approximately twofold higher cardiac protein expression, beating frequency, and amplitude. Further assessments in infarcted rats revealed that the infarct size and volume were almost half compared to the untreated groups, with elevated cardiac proteins and myocardial tissue development.

Bacterial cellulose (BC) is particularly interesting among nanocellulose from natural sources, because of its significantly higher purity. Whereas, plant cellulose contains traces of hemicellulose, lignin, pectin, and ash.^[^ ^79^ ^]^ Therefore, BC possesses no immunogenicity and a high crystallinity (84‐89%) compared to plant cellulose (40‐60%). BC also exhibits greater porosity, allowing higher water retention and better diffusion of biomolecules and their signals within the cellulose network. BC features a random interconnected network of nanofibers, closely mimicking the cardiac ECM in terms of morphology, fiber diameter, and pore size.^[^ ^80^ ^]^ In summary, BC is a strong contender for biomedical applications because of its superior mechanical properties, such as flexibility and tensile strength, biocompatibility, structural properties, such as porosity, and ECM‐like arrangements, hydrophilicity, and purity, compared to plant cellulose.^[^ ^79^ ^]^

**Figure 6:**
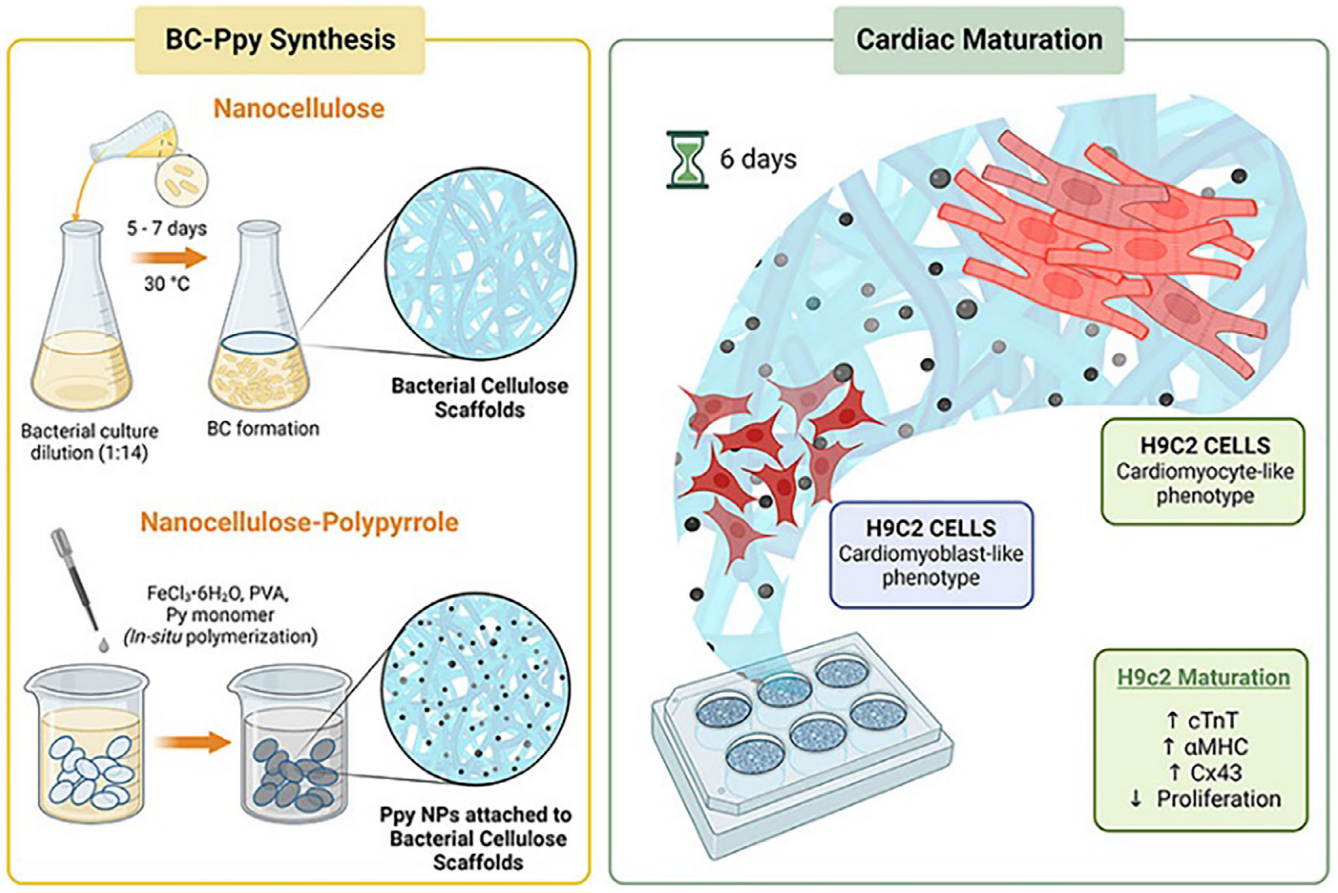
Schematic representation of BC‐Ppy nanofibrous scaffolds and their application as an in vitro platform for the differentiation of immature cardiac cells (H9c2), for future applications as cardiac patches for cardiac regeneration and healing. Reproduced with permission. ^[^ ^81^ ^]^ 2023, American Chemical Society.

Given the remarkable properties of BC, our research group has explored the BC‐Ppy NPs scaffolds as cardiac patches.^[^ ^81^ ^]^ We formulated BC: Ppy materials with distinct material properties by varying the Ppy NPs content in the composite. In this study, we conducted a comprehensive investigation into the design of BC: Ppy composites to achieve the desirable scaffold properties, and performed in vitro evaluation to assess their efficacy for cardiac therapies (**Figure** **6**). The BC‐Ppy scaffolds demonstrated excellent biocompatibility with cardiac fibroblasts (≈95%) and H9c2 cells (≈96%). Although BNCs are inherently biocompatible, incorporating Ppy NPs significantly enhanced cell viability (from ≈86% to ≈96%). Ppy NPs also markedly improved cell adhesion, morphology, and alignment. The beneficial effects of Ppy NPs can be attributed to enhanced cell‐cell communication facilitated by conducting nanoparticles. Our studies further revealed that Ppy NPs promoted the maturation of H9c2 cells into cardiomyocyte‐like phenotypes, as evidenced by the elevated expression of markers such as cardiac troponin T (cTnT), connexin 43 (CX43), and alpha‐myosin heavy chain (α‐MHC). Our research underscores the effectiveness of BC‐Ppy NPs in promoting cardiac differentiation without external stimuli, indicating significant promise as multifunctional patches for cardiac regeneration and healing.

In summary, many of these studies have not included evaluations in animal models or have only conducted biocompatibility studies in animals (**Table** **1**). It is essential that any nano‐biomaterial is thoughtfully designed, in view of the intended application, which, in this context, pertains to arrhythmia treatments. Therefore, it would be highly beneficial to conduct a comprehensive investigation on biocompatibility, therapeutic efficiency, biodegradation or elimination, and pharmacokinetics/pharmacodynamics through in vitro and in vivo evaluations. This would help establish their safety and efficacy, facilitating their effective application in the market.

**Table 1:** Studies using conducting nanomaterials for cardiac therapies. As we can see, there is a surging research interest in making use of conductivity for cardiac healing, with several novel nanomaterials gaining attention.

Material	Study	Key Findings	Advantages	Limitations
**Carbon Nanotubes (CNTs)**	CNTs in GelMA matrix.^[^ ^38^, ^39^ ^]^	Enhanced cell adhesion, maturation, and electrical communication in neonatal rat cardiomyocytes. Protected against cardio‐toxic drugs and cardiac inhibitors.	Presence of CNT improved mechanical & electrical properties.	Potential cytotoxicity, poor solubility in aqueous environments.
	a) CNT‐GelMA.^[^ ^40^ ^]^ b) CNT‐GelMA, compared with non‐conducting GO‐GelMA and conducting rGO‐GelMA.^[^ ^41^ ^]^	Increased intracellular Ca^2+^ transients, enhanced cell‐cell coupling, and improved gap junction formation due to CNTs.	Improves electrical conduction between cardiomyocytes. CNT‐GelMA has the most ECM‐like microenvironment.	Lack of evaluation of the materials in vivo.
	Gelatin‐coated bamboo‐like CNT forests.^[^ ^42^ ^]^	Gelatin coating enhanced the conductivity by 100%. Scaffolds preserved cytoskeletal integrity in fibroblasts. Enhanced contraction and communication in cardiomyocytes.	3D structure mimics native cardiac ECM, enhances signal propagation.	Potential inflammation due to CNT aggregation.
	BADSCs stacked with CNTs containing electrospun PCL/SF nanofibers.^[^ ^43^ ^]^	Promoted cardiomyocyte differentiation, cell motility, and vascularization.	Enhanced cardiomyogenesis and myocyte regeneration.	Large animal and human‐relevant models need to be evaluated.
**Gold Nanoparticles (AuNPs)**	Sprayable Peptide‐functionalized AuNPs.^[^ ^46^ ^]^	Improved cardiac strain force, ECG signal profile, and reduced infarct size (∼10% vs 60% untreated).	Sprayable formulation enabled smaller dosages, reduced off‐target toxicity.	Role of conductivity of AuNPs and exact mechanism of action were not studied.
	PEI coated AuNPs with PL in collagen hydrogel.^[^ ^26^ ^]^	Early onset of synchronous contractions in cardiomyocytes, promotes cardiac healing.	Synergistic effects of drug, AuNPs, and collagen,	Effect on animal models must be investigated.
	GNRs in GelMA vs SNP in GelMA scaffolds for hECT.^[^ ^48^ ^]^	GNR facilitated sarcomere development and organization. Enhanced electrical coupling between cardiomyocytes.	Elucidated the role of conductivity by comparing with a non‐conducting material (SNP).	Similar comparative studies in vivo can provide deeper understanding of role of conductivity.
**Polyaniline (PANI)**	PANI‐PGS‐PVA electrospun scaffolds.^[^ ^53^ ^]^	H9c2 cell elongation & alignment along fiber direction.	Compared the effect of scaffold fiber alignment on cardiomyocytes behavior. Tunable mechanical strength.	Limited solubility, potential inflammatory response. Only the Biocompatibility test is performed in vivo.
	PCL‐Gelatin nanofibers with PANI NPs.^[^ ^55^ ^]^	High cell adhesion, spreading, and proliferation of murine atrial cardiomyocytes.	Stimulation of beating and its propagation across cardiomyocytes, anti‐inflammatory ability.	Requires additional modification for biodegradability. In vivo evaluation is needed.
	PANI NPs on nanofibrous PCL mats with AA as dopant.^[^ ^54^ ^]^	Reduced intracellular levels, prevented oxidative stress caused by H_2_O_2_ and hypoxia damage.	A novel biocompatible and antioxidant dopant was used. Studies on oxidative stress and hypoxia conditions have been reported. Material showed promise in wound healing in vivo.	Further investigation on beating properties, arrhythmia conditions must be performed.
**Poly (ethylenedioxy thiophene)** **(PEDOT)**	PEDOT coated on SS stents, with GO, HEP, or PSS additives.^[^ ^59^ ^]^	Improved hemocompatibility, reduced protein adsorption, platelet adhesion, & delayed coagulation.	Unique anti‐fouling and anti‐clotting ability, suitable for stent coatings.	Biocompatibility was performed in vivo and only cell behavior was studied in vitro. Performance of actual stent coatings not evaluated.
	Injectable PEDOT: PSS collagen hydrogel.^[^ ^61^ ^]^	Enhanced conduction, calcium handling, & structural integrity. Prevented VT incidence and enhanced cardiac function in vivo.	Injectable formulation, promotes synchronous contractions. In vivo VT preventing ability is reported.	Possible immune response, although not significantly noted. Warrants large animal and patient‐relevant models for translation.
**Polypyrrole (PPy)**	Electrospun PCNU with Ppy NPs.^[^ ^71^ ^]^	Promoted cardiomyocyte alignment, synchronized contractions, and resolved atrial fibrillation in rat hearts.	Resolved arrhythmias in in vivo rat models.	Long‐term degradation of PCNU may release toxic byproducts.
	PPy‐grafted gelatin‐xanthan gum hydrogel.^[^ ^78^ ^]^	Improved conduction velocity, reduced scar size, increased vessel density, and decreased inflammation.	Injectable, biocompatible, enhances myocardial repair, prevents arrhythmias.	Lacks native ECM‐like microstructure.
	Tunicate sea squirt‐derived cellulose‐Ppy scaffold.^[^ ^75^ ^]^	Improving cardiac protein expression, beating frequency, and amplitude. Reduced infarct size in MI rat models.	Natural‐derived cellulose enhances biocompatibility and reduces immune response.	Requires additional functionalization to mimic cardiac ECM.
	BC‐Ppy scaffolds.^[^ ^81^ ^]^	BC exhibited higher purity, flexibility, and porosity compared to plant cellulose, mimicking cardiac ECM. Conductivity and biocompatibility are enhanced with Ppy integration. Ppy promoted maturation of hiPSC‐CMs for cardiac regeneration and healing.	Highly porous, mimics cardiac ECM, enhances electrical conduction.	Requires further research on calcium transients, beating frequency, and cardiac function in vivo.

## 5 Bio‐Evaluation Methodologies

CA can occur due to genetic abnormalities in a myriad of genes, or developmental disfunctions of components of the heart. Therefore, producing disease‐specific models for each case is complex and expensive. Different animals are recommended for different types of arrhythmias, further increasing the demand for infrastructure and technologies.^[^ ^82^ ^]^ This results in a more common use of 3D cell cultures and stem cells, leading to several proof‐of‐concept studies using nanomaterials for cardiac therapeutic applications, but evaluated only in vitro, with rare studies on animal models.

### 5.1 In Vitro Models

The initial toxicity and efficacy of nanomaterials in CTE are generally performed in 2D cell culture with cell lines such as isolated rat primary cardiomyocytes, immortalized cell lines derived from embryonic rat cardiac tissue (H9c2 cells),^[^ ^83^ ^]^ immortalized, spontaneously contracting mouse cardiomyocyte cell lines (HL‐1 cells) and proliferating, human cardiomyocyte cell line derived from primary adult ventricular tissue (AC16),^[^ ^84^, ^85^ ^]^ and human induced pluripotent stem cell‐derived cardiomyocytes (hiPSC‐CM).^[^ ^86^ ^]^ Given the heart's complex tissue organization, consisting of various cell types and microstructures, evaluating materials in a more representative model is crucial. In this aspect, co‐culturing multiple cell types is not possible in 2D cell cultures, affecting their similarity to the actual scenario in cardiac tissue structure and microenvironment.

**Figure 7:**
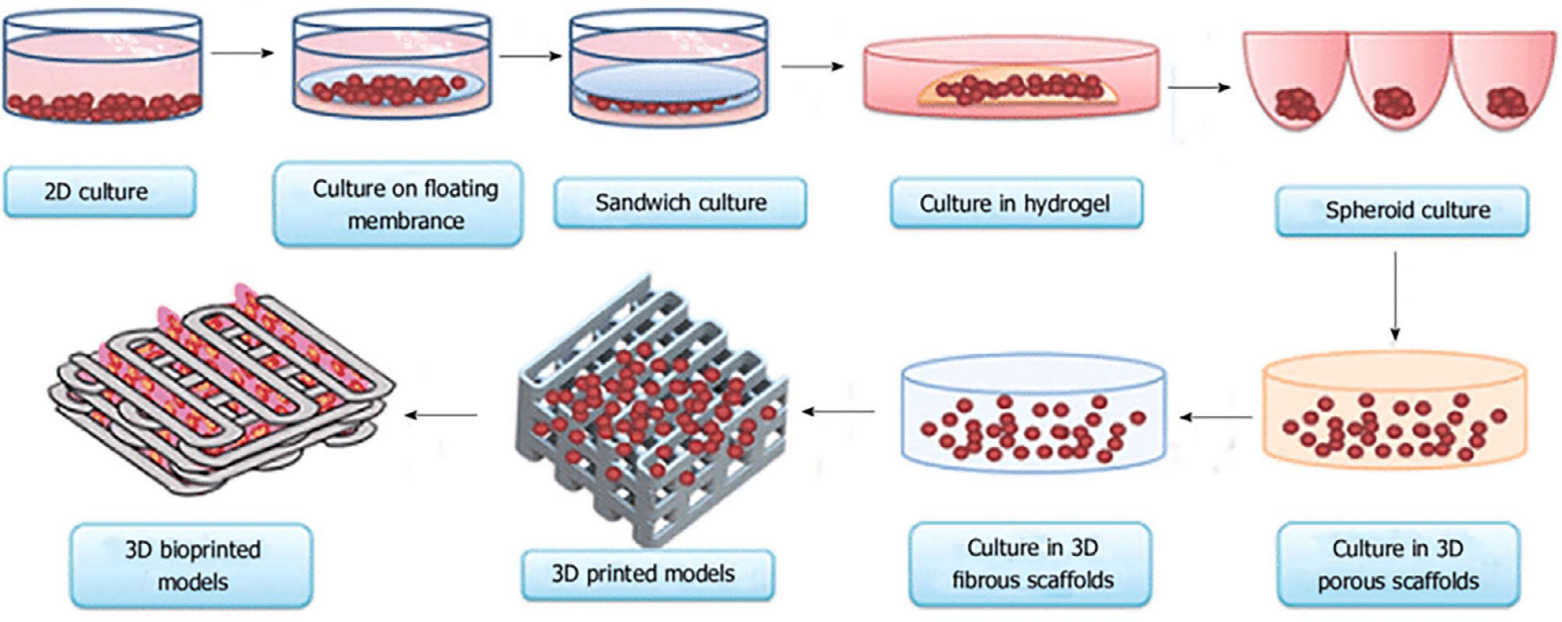
Overview of existing 2D and 3D cell culture models for in vitro evaluation of nanomaterials in cardiac cells and tissues. Reproduced with permission. ^[^ ^91^ ^]^ 2017, Baishideng Publishing Group Inc.

Recently, researchers have also been investigating 3D cell culture and organ‐on‐a‐chip microfluidic systems. Such systems are modelled to resemble several cardiac functions which are otherwise not represented in traditional cell cultures, including contractility, conduction velocity, and vasculature.^[^ ^87^, ^88^ ^]^ Generally, cell culture models are made 3D via two ways: i) by using a hydrogel scaffold with cardiac cells to form contractile engineered heart tissue (EHT), or ii) Spheroids formed through self‐assembly without the use of scaffold matrices.^[^ ^89^ ^]^ Recently, such free‐standing spheroids comprising a mixed population of cell types, such as cardiomyocytes, fibroblasts, stem cells, and endothelial cells, have gained a surging research interest.^[^ ^90^ ^]^ Additionally, 3D systems are frequently designed with micropatterns, microfluidics, and perfusions to mimic the functionalities of the human heart (**Figure** **7**).^[^ ^89^, ^91^ ^]^

Using a combination of nanofibrous scaffolds and neonatal rat ventricular cardiomyocytes (NRVMs) or human induced pluripotent stem cells derived cardiomyocytes (hiPSC‐CMs), MacQueen et al.^[^ ^92^ ^]^ developed 3D models of the left ventricle of the human heart. The nanofibrous scaffolds enabled the shape and assembly of the tissue constructs. These ventricular models exhibited a diastolic chamber volume of ≈ 500 µL, similar to native rat ventricles. The cardiomyocyte spontaneous beat rates in the models were found to be similar to those in other in vitro assays based on NRVMs or hiPSC‐CMs. However, parameters such as ejection fraction and contractility were significantly smaller than their corresponding values in rodents and humans. A more recent advancement in the development of 3D ventricular heart models is that, in addition to mimicking the myocardium's structural complexity, researchers have also developed models mimicking the heart's functional dynamics. For instance, the heart's helical myocardial structure enables a unique pumping function through a twisting motion of the heart that facilitates efficient ejection from the ventricles. This twisting motion is supported by the helical ventricular myocardial tissue structure, where the ventricular myocardium is made of a single, continuous, helical muscle band that twists around the heart's core. Bearing this in mind, Williams et. al.^[^ ^93^ ^]^ produced 3D anisotropic ventricular tissues with organized cellular architecture using nanopatterned cell‐sheet engineering techniques. In this study, the authors assembled multilayered, patterned cardiac sheets on a 3D printed conical mold to result in a helical transmural architecture. The cardiomyocytes aligned parallel or perpendicular to the long axis showed improved contractile pressures, compared to angled or randomly oriented cells. Interestingly, the cardiomyocytes in perpendicular sheets underwent spontaneous remodeling to parallel alignment in a span of 4 days. The authors reported that the cells undergo remodeling to reduce local shear stress. Thus, this model demonstrates that anisotropic myocardial organization provides functional advantages over isotropic models. The model further elucidates the functioning of helical myocardial patterning and how mechanical signals can aid in cell and tissue remodeling and the relationship between myocardial structure and function. Such advancements in 3D ventricular models pave the way for complex myocardial architectures that can fully mirror human cardiac structure and function.

### 5.2 Large Animal Models

The common laboratory animals, such as rodents, rabbits, pigs, and dogs, are the usual models for arrhythmia research.^[^ ^94^, ^95^ ^]^ An ideal model should exhibit structural, genetic, and functional similarities to the human heart, cost‐effective maintenance, and allow precise genetic modifications.^[^ ^96^ ^]^ However, no single animal model fulfills all the criteria for arrhythmia research. Currently, animal models are chosen based on the aspects of cardiac rhythm or the type of arrhythmia being investigated. For example, although mice and rats are popular models, their cardiac electrophysiology significantly differs from that of humans, making them less suitable for electrophysiological studies but appropriate for genetic research, arrhythmias induced by myocardial ischemia, such as atrial and ventricular fibrillation, and tachycardia.^[^ ^97^, ^98^ ^]^ Rabbit models with induced MI have been reported, but they did not exhibit any signs of arrhythmia.^[^ ^99^ ^]^ Similarly, while ischemia can be induced in dogs and has been used in the past, their use has declined because of the necessity of pacemaker implantation to observe significant phenotypes. Conversely, pig models are advantageous for studying ischemia‐induced arrhythmias and ventricular ion channel dysfunction due to their phenotypic effects resembling humans, anatomical similarity, and the feasibility of producing reproducible infarct sizes.^[^ ^100^ ^]^

Whereas for investigating atrial fibrillation, tachypacing of goat hearts was carried out. This study revealed substantial information that persistent atrial fibrillation leads to morphological changes in cardiomyocytes and myocardial dedifferentiation.^[^ ^101^ ^]^ Interestingly, in addition to goats, all other animal models except rodents, i.e., rabbits, dogs, pigs, and sheep, are also tachypaced for atrial fibrillation‐related research.^[^ ^96^ ^]^ Notably, dogs and pigs exhibit electrophysiological characteristics more closely aligned with those of humans than goats, with pigs being the most cost‐effective option. Consequently, although goats have been traditionally studied, pigs may soon emerge as a viable alternative. Whereas in the case of ventricular tachycardia and its associated heart failure, rats, dogs, and pigs are popular choices. In addition to the type of arrhythmia, the causative factor also plays a crucial role in selecting an animal model. For instance, while arrhythmia induced by alcohol is commonly studied in pigs, rats are preferred in the case of arrhythmia caused by pulmonary hypertension.^[^ ^102^, ^103^ ^]^ Rabbits, goats, and pigs show similar electrophysiology and molecular pathways to humans, respectively. Therefore, mutant strains of these animals are employed to study arrhythmias at the genetic and molecular level. Potassium channel mutations (KCNQ1 and KCNH2) in rabbits serve as an ideal model for studying repolarization, TGF‐β1 mutated goats for atrial fibrillation, and SCN‐5A mutated pigs for Brugada syndrome.^[^ ^104^, ^105^, ^106^, ^107^ ^]^ Rats and mice are generally popular candidates for initial in vivo evaluations, as they are easier to use in terms of availability, cost, ease of handling and maintenance, and better ethical processing. Although rodents provide reliable insights for diseases like cancer, the digestive and nervous systems, they are not optimal models for cardiac diseases because of their differences from the human heart, further augmenting the need to use larger animals, even for initial evaluations, to provide reliable information. However, as we have seen thus far, the animal model needed is unique for each type of arrhythmia and the underlying cause, and creating genetically modified large animals is complex, costly, and generally discouraged at the early research stages. Additionally, these animals have lower reproductive rates and long gestational periods, resulting in a reduced reproductive yield even when mutant strains are produced.

### 5.3 Alternative Small Animal Models

Small animal models, such as zebrafish, Drosophila melanogaster, and Caenorhabditis elegans, to assess the toxicity and efficacy of pharmaceuticals and nanomaterials during early developmental stages are increasingly gaining attention, since they facilitate the application of the 3Rs (reduce, reuse, and recycle), produce data on large populations at statistically significant levels, and expedite the progress of novel candidates toward large animal evaluations.

#### 5.3.1 Zebrafish

**Figure 8:**
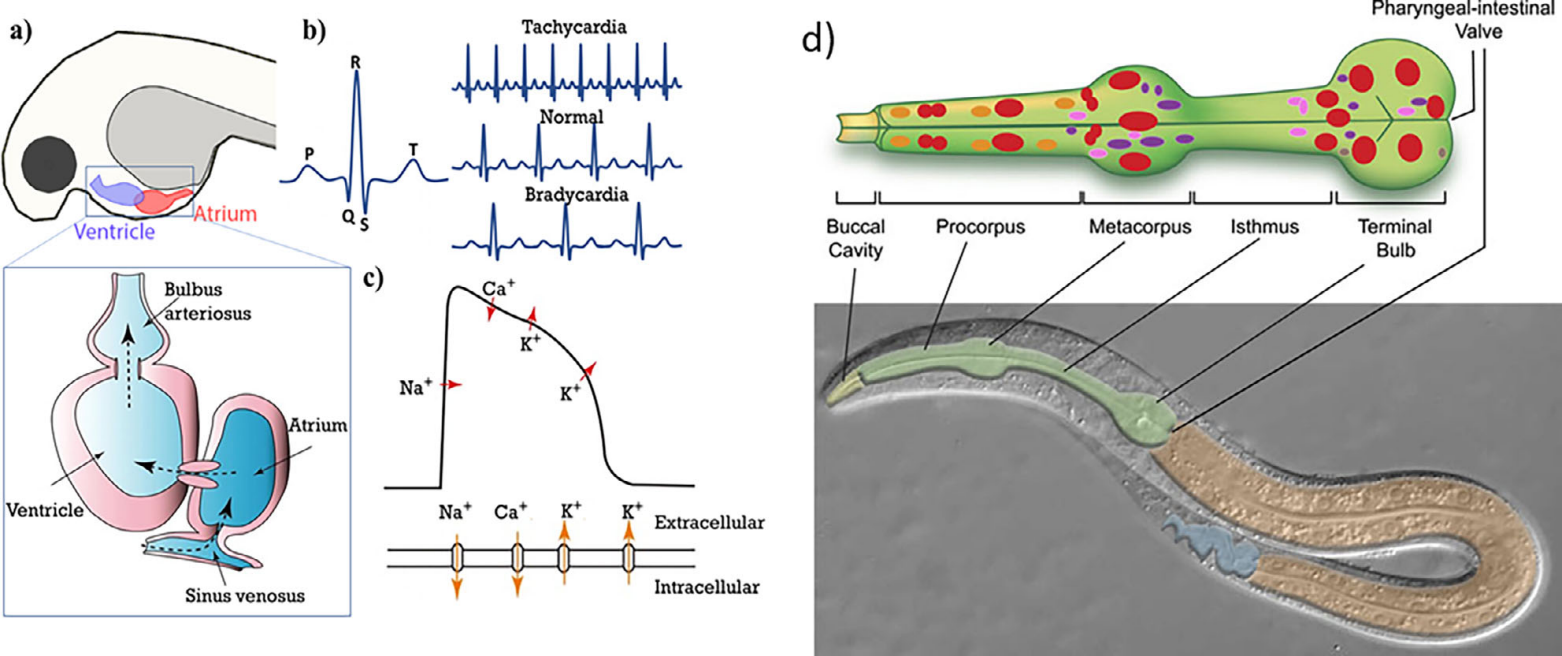
a) Heart of the zebrafish with two chambers: one atrium and one ventricle. b) Zebrafish heart ECG in normal and arrhythmic conditions. c) An action potential with different phases marked by ion transfer. d) The pharynx of *C. elegans* with the detailed cell and tissue organization: red: muscles, purple‐ neurons, orange‐ epithelia, pink‐ marginal cells, and brown‐ glands. Reproduced with permission. ^[^ ^11^ ^]^2023, UAB, and ^[^ ^137^ ^]^ 2007, WormBook Research Community.

Zebrafish (*Danio rerio*) are fish models that are transparent at the larval stage and have a high reproductive rate. In contrast to human hearts, zebrafish hearts are made of only two chambers: a single atrium and ventricle each. Despite this simplicity, the CCS of zebrafish and humans have been found to be closely related: i) pacemaker cells in zebrafish are located at the junction of the sinus venosus and atrium, analogous to the human pacemaker cells present at the SAN, ii) cardiomyocytes present at the AVN region are slow‐conducting, mimicking the AVN cardiomyocytes of the human heart^[^ ^108^, ^109^ ^]^(**Figure** **8**a), iii) the zebrafish heart rate ranges from 120 to 180 beats per minute (bpm), a heart rate more akin to that of humans (60–90 bpm) compared to other animals, iv) the phases of cardiac cycle, represented by the ECG and action potential, were also similar to those in humans (Figure 8b,c), and finally v) some of the cardiac ion channels of zebrafish and humans share genetic orthology. Genetically modified zebrafish strains with mutant ion channels are popular as arrhythmia models to study ion channel dysfunctions and drug actions.^[^ ^110^, ^111^ ^]^

Owing to these advantages and similarities, zebrafish larvae are being utilized as a reliable small‐animal model for investigating cardiac physiology, cardiotoxicity, and anti‐arrhythmic effects of drugs in recent years.^[^ ^108^, ^112^ ^]^ Cornet et al. studied the cardiac effects of an array of 25 compounds by evaluating the heart rate and frequency of zebrafish larvae after exposure to these candidates. Their findings indicated that the effect of drugs on zebrafish larvae corresponded to the effects reported for humans, achieving a 68% true positive rate (cardiotoxic drugs affecting heart rate) and an 89% true negative rate (non‐toxic drugs not affecting heart rate), surpassing the results obtained with human‐induced pluripotent stem cells (hiPSC‐CMs) and rodents.^[^ ^113^ ^]^ Building on this work to facilitate large‐scale application of zebrafish testing, the same researchers developed Zecardio – a combined hardware and software platform offering high‐throughput screening and high‐resolution analysis.^[^ ^114^ ^]^ The authors evaluated 92 compounds with reported cardiotoxicity using zebrafish and Zecardio, and compared the results with hiPSC‐CMs. The study found that zebrafish exhibited greater sensitivity and 30% higher true positive rate than hiPSC‐CMs. The researchers attributed this to the complexity of the complete organism, wherein absorption, distribution, metabolism, and excretion (ADME), adrenergic signaling, and ion‐channel mechanisms of the zebrafish model, which closely resemble those of humans, compared to hiPSC‐CMs largely affect the pharmacokinetics and pharmacodynamics of the tested drugs.

The zebrafish embryo and larvae are optically transparent, allowing direct visualization of nanomaterial biodistribution, uptake, and clearance using microscopy, avoiding invasive procedures. Additionally, several advantages, including the rapid development from embryo to larvae (48‐72 h), high reproductive rate (up to 300 eggs per week), and the possibility of water‐borne exposure of nanomaterials, have led to the positioning of zebrafish as one of the leading small animal models for nanomaterial evaluation.^[^ ^115^, ^116^, ^117^ ^]^ Zebrafish models have also been reported specifically for the evaluation of nanoparticles (NPs) for cardiac therapies. In a recent work,^[^ ^118^ ^]^ nitric oxide (NO) releasing chitosan hydrogel NPs (NO‐RPs) incorporating iron oxide NPs (SPIONS) were studied in zebrafish larvae models, for alleviating heart failure induced by a reduced nitric oxide bioavailability. In this work, 24 hours post‐fertilization zebrafish embryos were utilised, with aristolochic acid‐induced heart failure. The authors measured several parameters, such as heart beat, diameter, blood flow velocity, and shear stress in major blood vessels, the primordial cardinal vein and dorsal aorta (PCV &DA), and cardiac output, to assess the cardiac function of injured embryos versus after exposure to the NO‐RPs for 48 h. The NO‐RPs improved the survival rate of the heart failure zebrafish model, restored the reduced heartbeat, improved the diameter, slowed the blood flow velocities in the DA and PCV vessels, and improved cardiac output in the injured embryos. Furthermore, mRNA expression levels of pro‐inflammatory markers IL‐6 and COX‐2 were significantly downregulated in NO‐RPs treated embryos, suggesting reduced inflammation. Thus, using zebrafish models allowed the researchers to measure cardiac output, blood vessel parameters, shear stress, etc., which is not possible with in vitro models, highlighting the importance of employing small animals prior to advanced phases in such studies. Interestingly, cryoinjury‐induced MI models as well as drug‐induced arrhythmia models are well established in zebrafish.^[^ ^119^ ^]^ Although NPs‐mediated drug or gene delivery for cardiac therapies is investigated, to date, no published work directly tests NPs with anti‐arrhythmic ability, or biomaterials as cardiac patches on such zebrafish models. A promising future direction would be to utilize zebrafish models for evaluating the efficacy of micro‐scale biomaterials or injectable hydrogels as cardiac patches, or the components/additives of cardiac scaffolds – such as a drug or nanoparticle for their efficacy in MI‐induced arrhythmia treatment to obtain informative real‐time cardiac parameters in a high‐throughput, rapid manner, which would be much closer to large animal models and the human heart. However, due to their small size, cardiac patches cannot be tested in their macroscopic full‐implantable form; therefore, it is difficult to assess their mechanical performance in the heart that can be translatable to humans. Additionally, zebrafish myocardium is naturally regenerative after injury – recovering within weeks, making them unsuitable to evaluate chronic post‐MI remodeling.

#### 5.3.2 Drosophila Melanogaster

The fruit fly (Drosophila melanogaster) is an invertebrate model. The Drosophila heart is homologous to the human heart in the functional and developmental aspects, particularly the sarcomere.^[^ ^120^, ^121^, ^122^ ^]^ Drosophila's short life cycle of 10 days at 25 °C, high reproductive rate of 2000 eggs per female, low maintenance costs, and conservation of genes and cellular pathways make it an efficient model for rapid evaluation. Notably, Drosophila shares genetic homology with 77% of the genes associated with human diseases, including 26 genes associated with CVDs.^[^ ^123^ ^]^ The Drosophila heart is composed of a linear tube, akin to an embryonic heart tube in vertebrates, and many regulatory genes related to cardiac development, function, aging, and signaling pathways, such as NK‐2, MEF2, GATA, Tbx, and Hand, are conserved evolutionarily.^[^ ^124^ ^]^ Consequently, Drosophila offers significant advantages for manipulating gene expression and identifying the genes and mechanisms involved in cardiac development, pathology, and pathophysiology. Much like zebrafish, the drosophila's ECG also share high similarity with humans, leading to their application in studying cardiomyopathies and channelopathies. Crucial cardiac functions, namely, pumping and muscle contractions, are tightly regulated by calcium signaling, which involves proteins such as voltage‐gated Ca^2+^ channels (VGCC), ryanodine receptors (RyR), and the sarcoendoplasmic reticulum calcium ATPase (SERCA) pump. RyR and SERCA channel mutations have been reported to cause arrhythmia in Drosophila cardiac models.^[^ ^122^, ^125^ ^]^

The voltage‐gated K^+^ channels of Drosophila, such as KCNQ, are orthologous to human K^+^ channels, enabling their wide use as channelopathy models with potassium channel mutations,^[^ ^126^ ^]^ and they also stand as a promising candidate for studying class III anti‐arrhythmic drugs. One such example is celecoxib, an NSAID (non‐steroidal anti‐inflammatory drug), which targets voltage‐gated K^+^ channels, and was found to induce arrhythmia in the heart of drosophila larvae.^[^ ^127^ ^]^ Furthermore, in the case of Drosophila, extensive and in‐depth analysis has been enabled through techniques such as optical coherence tomography for disease phenotyping and powerful electrophysiological, mechanical, and histological tools to characterize cardiac development, tissue properties, and structure, making them an ideal model for understanding cardiac development, aging, and genetic and molecular cardiac function.

Drosophila models are generally more leveraged for understanding the genetic mechanism of cardiac arrhythmia and metabolic cardiomyopathy. Drug intervention studies, such as the effect of antioxidants on high‐fat diet‐induced arrhythmia, and the effect of metformin on high‐sugar diet‐induced cardiac dysfunction, have been reported.^[^ ^128^, ^129^ ^]^ However, Drosophila has not yet been explored for nanomaterial or biomaterial therapies for cardiac arrhythmia. Therefore, an interesting way forward would be to utilize Drosophila for high‐throughput testing of cardioprotective nanomaterials, nanoparticles carrying antioxidants, ion‐channel modulators, anti‐arrhythmic drugs, or metabolic regulators in arrhythmia models such as KCNQ mutants. While cardiac patches can't be implanted in flies, nanoparticles that improve electrical conduction (e.g., conductive nanoparticles) could be systemically administered, and their effects on arrhythmia index (heart rate variability, conduction velocity, etc.) could be studied, especially in ion‐channel mutants. Drosophila has unique strengths, however there are also clear limitations – they have an open circulatory system, lacking a coronary vasculature; therefore, a true myocardial infarction cannot be modeled.^[^ ^130^ ^]^ Additionally, flies lack organs such as liver and kidneys, therefore, nanoparticle distribution, clearance, metabolism, and organ targeting are also largely different than humans. Therefore, while drosophila can be beneficial for high‐throughput early screening of candidates especially for genetic studies, they have limitations in assessing more translatable cardiac and toxicity parameters.

#### 5.3.3 Caenorhabditis Elegans

*Caenorhabditis elegans* (*C. elegans*) is a free‐living transparent nematode that has recently been explored as an alternative model for CA. Interestingly, among small animal models, *C. elegans* was the first to have a sequenced genome (in 1998) as well as a completed connectome (in 2019).^[^ ^131^ ^]^ *C. elegans* are short living (≈ 3–4 weeks), possess a rapid and high reproduction rate (≈ 300 (in 72 h) – 1400 per adult), optical transparency, and are feasible to store at −20 °C, facilitating long‐term preserving of mutant strains. *C. elegans* also offers a set of distinctive advantages: the creation of mutant strains is straightforward through nutritional mutagenesis (by feeding mutagens) or genome editing tools such as CRISPR‐Cas9 for site‐specific mutations. Owing to the storage ability, simple mutagenesis, and large reproduction rate, a large‐scale production of mutant strains is possible in a short period. Consequently, *C. elegans* is a popular small‐animal model in biomedical research, especially for evaluating nanomaterials for various factors, namely, toxicological evaluations on a large scale, to identify molecular targets of the nanomedicines, the pharmacokinetics (dissolution/accumulation), and pharmacodynamics (ADME).^[^ ^132^, ^133^ ^]^ The worms possess a pharynx (Figure 8d), a continuously pumping organ responsible for grinding food and their progression toward the intestine.^[^ ^134^ ^]^ The pharynx comprises electrically coupled muscle cells, which are continuously contracting and relaxing, similar to the human heart. The electrical coupling across muscle cells is facilitated through gap junctions at the cell membranes, consisting of VGCC, which are responsible for action potential propagation from one cell to the next, resulting in muscle contraction.^[^ ^135^, ^136^ ^]^

**Figure 9:**
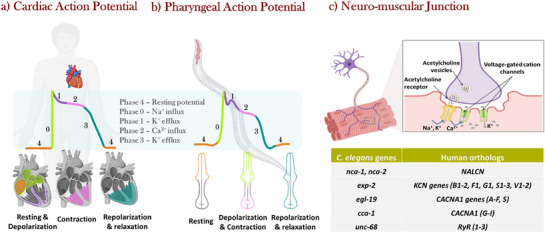
Representation of a) humans and b) *C. elegans* actional potential phases, along with the muscle contractions and c) ion transfer events occurring at the neuromuscular junction. Reproduced with permission. ^[^ ^141^ ^]^ 2023, American Chemical Society.

The pharyngeal action potential resembles the human cardiac action potential, wherein a sequence of Ca^2+^‐mediated membrane depolarization occurs, resulting in contraction, followed by repolarization, and finally the resting state before the next action potential cycle^[^ ^137^ ^]^ (**Figure** **9**). This cycle of depolarization and repolarization is regulated by the transport of K^+^ and Ca^2+^ ions through K^+^, and voltage‐gated Ca^2+^ channels (L‐type, and T‐type). Notably, mutations in these ion channels can lead to dysregulation of muscle contraction, manifesting as altered pharyngeal pumping, similar to how ion channelopathies in the heart can result in CA.^[^ ^138^, ^139^ ^]^ Interestingly, the Na^+^, K^+^, and Ca^2+^ channels of *C. elegans*, such as nca‐1 and nca‐2 (Na^+^), exp‐2 (K^+^), egl‐19, cca‐1 (Ca^2+^), and unc‐68, are genetically homologous to the human NALCN (Na^+^), KCNQ1 (K^+^), CACN1 (Ca^2+^), and RyR2, respectively.^[^ ^140^ ^]^

Researchers have genetically modified *C. elegans* to create disease models in the last few years and have found that they display arrhythmia‐specific traits. The *egl‐19* gene of *C. elegans* is homologous to CACNA1A in humans, which encodes the Cav1.2 channel, an L‐type voltage‐gated Ca^2+^ channel. While Cav1.2 channel mutations result in long QT syndrome in humans, homologous mutations in *egl‐19* also induced long QT‐like syndrome in *C. elegans*, with phenotypes like prolonged pump duration and impaired ability to pump at high frequency.^[^ ^142^ ^]^ Similarly, mutations in human Ca^2+^ channels orthologs *csq1* and *unc‐68* altered the pharyngeal pumping, resulting in missed pumps, either at half frequency or without pumping. Remarkably, treatment with the anti‐arrhythmic drug benzothiazepine restored normal pharyngeal pumping in worms with these mutations.^[^ ^143^ ^]^

The presence of arrhythmia‐inducing mutations in the heart, which lead to similar arrhythmia‐like phenotypes in *C. elegans*, strongly supports the use of *C. elegans* as a model organism for evaluating cardiotoxicity and the effectiveness of antiarrhythmic drugs and nanomaterials. In this aspect, we assessed the potential of *C. elegans* as a model for CA to evaluate anti‐arrhythmic drugs and promising anti‐arrhythmic conducting NPs.^[^ ^141^ ^]^ We investigated the pharynx of *C. elegans* as a platform for studying cardiac rhythm using two well‐known cardiac drugs, propranolol (PL) and racepinephrine (RE), which decrease and increase heart rates in humans, respectively. The effects of these drugs on pharyngeal pumping rates were consistent with their effects on human cardiac pumping rates, supporting the use of *C. elegans* for testing novel cardiac drugs and nanomaterials. Subsequently, we examined the pharyngeal effects of Ppy NPs. After 24 h of treatment, a 25% increase in the pumping rate was observed in Ppy NP‐treated worms compared to untreated worms. Additionally, calcium transient studies on wild‐type and cca‐1 calcium channel‐mutated *C. elegans* strains revealed the impact of NPs on intracellular calcium signaling as a potential mechanism of action of the conducting NPs, similar to the in vitro evaluations of BC‐Ppy scaffolds by our group, as well as other in vitro studies of Ppy NPs and other conducting NPs (**Table** **2**).

**Table 2:** Suitable animal models used in cardiac evaluations for different CVD conditions, according to each model's physiology, genetic similarity, and several other factors, along with their advantages and limitations.

	Animal	Conditions studied	Advantages	Limitations
**Conventional Animal Models**	**Mice & Rats** 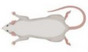	Myocardial ischemia‐induced arrhythmias (ventricular & atrial fibrillation, ventricular tachycardia), genetic studies, and pulmonary hypertension‐induced arrhythmias.^[^ ^97^, ^98^ ^]^	Cost‐effective, easy genetic modification, high reproduction rate, ideal for proof‐of‐concept studies.	Poor electrophysiological resemblance to humans.^[^ ^97^, ^98^ ^]^
	**Rabbits** 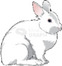	Genetic studies (potassium channel mutations for repolarization). Atrial fibrillation induced by atrial tachypacing. Potassium channel mutations to study repolarization.^[^ ^99^ ^]^	Ideal to study potassium channels, genetically modifiable, and better electrophysiological resemblance than rodents.	No reported arrhythmias in MI models.^[^ ^99^ ^]^
	**Dogs** 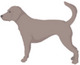	Previously used as ischemia models, ventricular tachycardia induced heart failures, atrial fibrillation induced by tachypacing.^[^ ^96^ ^]^	Closer electrophysiology to humans than goats.	Requires pacemaker for significant ischemic phenotype, ethical concerns.
	**Pigs** 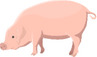	Ischemia‐induced arrhythmias, ventricular ion channel function studies, alcohol‐induced atrial fibrillation, and genetically modified models (SCN‐5A mutations for Brugada syndrome).^[^ ^100^, ^102^, ^103^ ^]^	Closest cardiac anatomy to humans, reproducible infarct sizes, cost‐effective compared to other large animals.	Expensive to maintain, complex genetic modification.
	**Goats** 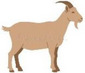	Atrial fibrillation (by tachypacing).^[^ ^101^ ^]^	Persistent atrial fibrillation model, morphological changes mimic humans.	Less electrophysiological resemblance to humans compared to pigs/dogs.^[^ ^96^ ^]^
	**Sheep** 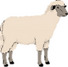	Atrial fibrillation.^[^ ^96^ ^]^	Similarities in cardiac morphology to humans.	Less widely studied compared to pigs and dogs.
**Alternate small animal models**	**Zebrafish** 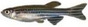	Cardiotoxicity and efficacy evaluation of drugs, ion channel mutations for arrhythmia models.^[^ ^110^, ^111^ ^]^	Transparent, high reproduction rate, similar heart rate to humans, genetic similarity.^[^ ^108^, ^112^ ^]^	Simplified heart structure (one atrium, one ventricle)
	**Drosophila melanogaster (Fruit fly)** 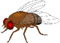	Cardiomyopathy, arrhythmias, heart development studies, and potassium channel mutation studies.^[^ ^122^, ^125^, ^126^ ^]^	77% genetic homology to human cardiac genes, low cost, short life cycle.	Primitive heart structure, lacks full circulatory system.^[^ ^130^ ^]^
	**Caenorhabditis elegans (*C. elegans*)** 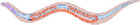	Arrhythmia studies, cardiotoxicity evaluation, and long QT syndrome models.^[^ ^141^, ^142^ ^]^	Genetic and ion channel homology to humans, simple and cost‐effective, high‐throughput screening potential.^[^ ^137^, ^138^, ^139^, ^140^ ^]^	Lacks a true heart, and vascular system. Pharyngeal pumping used as a surrogate for cardiac function.

## 6 Conclusion

CVDs remain the leading cause of death worldwide despite extensive efforts to develop new treatment methods. Conventional treatments, such as pacemakers and DC cardioversion, primarily target arrhythmias, MIs, and related heart failure; however, they fail to address the underlying causes. Acknowledging that these standard therapies often have significant contraindications and lack the necessary effectiveness and precision is imperative. Consequently, there is an urgent need for multifunctional therapies that offer high efficiency, biocompatibility, and minimal side effects to effectively treat and manage cardiac diseases in today's healthcare landscape. Intrinsically conducting nanomaterials are emerging as a beacon of hope in cardiovascular therapeutic research owing to their promising multifunctional properties, including biocompatibility, environmental stability, ease of synthesis, and unique electrical conductivity. Notably, the electrical conductivity of these materials holds immense potential for cardiac therapies by enhancing electrical coupling between cardiac cells, improving intercellular communication, facilitating cardiomyocyte maturation, and promoting cardiac regeneration and repair. However, addressing the lack of animal studies in the current literature on this subject is crucial. Despite the substantial potential demonstrated by a growing number of studies worldwide, there is a noticeable gap in the progression of materials to the later developmental stages. The scarcity of in vivo studies on conducting nanomaterials for cardiac therapies may stem from the challenges associated with developing animal models with the specific mutations of interest. Given that arrhythmia models often require larger animals, such as dogs and pigs, various factors, including a lack of expertise, inadequate housing facilities, funding challenges, and the need for ethical approval, may impede the advancement of innovative treatment options, such as nanomedicines. Conversely, small animals such as zebrafish, fruit flies, and *C. elegans* offer invaluable insights into arrhythmia research. These smaller models enable more accessible, rapid, cost‐effective, and large‐scale evaluations. Furthermore, they support the implementation of the 3Rs principle in biomedical research: Reduce, Reuse, and Recycle. This approach can effectively bridge the gap between initial in vitro evaluations and more comprehensive animal studies, paving the way for developing multifunctional and practical therapeutic nanomaterials for clinical use.

## Conflict of Interest

The authors declare no conflict of interest.
